# Mesenchymal Stromal Cells Play an Analgesic Role Through a *Npy2r* Sensory Neuron‐Mediated Lung‐to‐Brain Axis

**DOI:** 10.1002/advs.202504922

**Published:** 2025-08-28

**Authors:** Jing Huang, Taihe Zhou, Yueming Sun, Yuanchen Ma, Yiwen Deng, Keyu Chen, Ruijie Li, Yuan Qiu, Tao Wang, Xiaoyong Chen, Li Huang, Lu Zhu, Xueyi Tu, Ying Wang, Zheming Liu, Rouchen Lin, Yunli Tong, Yuantao Li, Heshe Li, Lin Nie, Rui Fang, Jianqi Feng, Xuhong Wei, Xia Feng, Andy Peng Xiang, Xiaoran Zhang

**Affiliations:** ^1^ Department of Anesthesiology The First Affiliated Hospital Sun Yat‐sen University Guangzhou Guangdong 510080 China; ^2^ Center for Stem Cell Biology and Tissue Engineering Key Laboratory for Stem Cells and Tissue Engineering Ministry of Education Sun Yat‐Sen University Guangzhou Guangdong 510080 China; ^3^ National‐Local Joint Engineering Research Center for Stem Cells and Regenerative Medicine Zhongshan School of Medicine Sun Yat‐Sen University Guangzhou Guangdong 510080 China; ^4^ Department of Physiology and Pain Research Center Zhongshan School of Medicine Sun Yat‐Sen University Guangzhou Guangdong 510080 China; ^5^ Jiangxi Provincial Key Laboratory of Cell Precision Therapy School of Basic Medical Sciences Jiujiang University Jiujiang Jiangxi 332005 China; ^6^ Center of Stem Cell and Regenerative Medicine Gaozhou People's Hospital Maoming Guangdong 525000 China; ^7^ Shenzhen Qianhai Shekou Free Trade Zone Hospital Shenzhen China; ^8^ Department of Histoembryology and Cell Biology Zhongshan School of Medicine Sun Yat‐Sen University Guangzhou Guangdong 510080 China

**Keywords:** analgesia, mesenchymal stem/stromal cells (MSC), Npy2r sensory neuron, vagal lung‐to‐brain axis

## Abstract

Mesenchymal stromal cells (MSC) have emerged as a promising therapeutic option for neuropathic pain (NP), but the mechanisms remain elusive. Using murine pain models, it is demonstrated that MSC effectively alleviates pain, with efficacy comparable to dexmedetomidine, a moderate analgesic. Mechanistically, peripheral delivery of MSC‐activated pulmonary *Npy2r*‐expressing vagal sensory neurons, which project to the nucleus tractus solitarius and ventral lateral periaqueductal gray area, drives analgesia via the vagal lung‐to‐brain pathway. Chemogenetic activation of *Npy2r* sensory neurons similarly ameliorates spared nerve injury (SNI)‐induced mechanical allodynia and thermal hyperalgesia. Furthermore, it is found that MSC‐derived extracellular ATP, released via pannexin1, activates *Npy2r* sensory neurons through purinergic receptor P2X2 (P2rx2). Strikingly, inhalation of a P2rx2 agonist produced significant therapeutic effects in SNI mice. Together, these findings reveal that *Npy2r* sensory neuron‐mediated lung–brain axis underlies MSC‐induced analgesia and highlight the potential of targeting body–brain pathways for novel NP treatments.

## Graphical abstract

Mesenchymal stromal cells (MSC) are considered a promising alternative for neuropathic pain (NP) treatment, but underlying mechanisms remain elusive. Huang et al. illustrate that a *Npy2r* sensory neuron‐related lung–brain axis contributes to MSC analgesia. These findings provide insights into aiming the body–brain axis to develop new strategies for NP therapy.

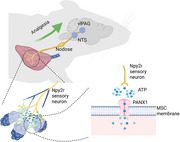

## 1 Introduction

Neuropathic pain (NP), a debilitating condition caused by somatosensory nervous system lesions or diseases, remains a major global health challenge^[^ ^1^ ^]^ Characterized by abnormal pain perception and negative emotions,^[^ ^2^ ^]^ NP severely impairs quality of life, often leading to anxiety, depression, sleep disturbances, reduced mobility, and cognitive decline when poorly managed.^[^ ^3^ ^]^ Current pharmacologic therapies provide inadequate relief and are frequently limited by adverse effects during prolonged use.^[^ ^4^ ^]^ Widely prescribed non‐steroidal anti‐inflammatory drugs (NSAIDs) like aspirin and ibuprofen, while effective against pain and inflammation, pose risks of gastrointestinal bleeding and cardiovascular complications.^[^ ^5^ ^]^ Opioid analgesics, though effective for moderate‐to‐severe pain, carry addiction potential and are unsuitable for chronic use.^[^ ^6^ ^]^ Adjunctive non‐pharmacological interventions, including percutaneous electrical nerve stimulation, spinal cord stimulation, and acupuncture, show variable efficacy due to individual heterogeneity.^[^ ^7^ ^]^ Given these limitations, developing targeted, disease‐modifying analgesics for NP represents a critical unmet medical need.

Mesenchymal stromal cells (MSC) are multipotent adult stem cells with remarkable regenerative capacity, immunomodulatory properties, and paracrine functions. These cells can be isolated from various tissues, including bone marrow, adipose tissue, and umbilical cord.^[^ ^8^ ^]^ Extensive preclinical studies have demonstrated MSC efficacy in alleviating diverse pain conditions, including nociceptive, neuropathic, and cancer‐related pain. Clinical trials further support their therapeutic potential for chronic pain management.^[^ ^9^ ^]^ However, treatment outcomes appear variable, influenced by factors such as MSC dosage, tissue source, patient characteristics, and disease severity.^[^ ^10^ ^]^ The mechanisms underlying MSC‐mediated analgesia remain incompletely understood. While some studies attribute their effects to extracellular vesicle‐mediated anti‐inflammatory actions,^[^ ^11^ ^]^ others suggest direct modulation of peripheral sensory neurons.^[^ ^12^ ^]^ Emerging evidence highlights the importance of body–brain communication in pain regulation,^[^ ^13^ ^]^ with peripheral sensory neurons relaying visceral state information to the central nervous system (CNS).^[^ ^14^ ^]^ Notably, we recently demonstrated that intravenously administered MSC primarily engraft in the lungs, where they interact with pulmonary sensory neurons and initiate central nervous system signaling.^[^ ^15^ ^]^ Based on these findings, we propose that pulmonary sensory innervation plays a crucial role in MSC‐induced pain relief in neuropathic pain models.

The vagus nerve serves as a critical body–brain conduit, comprising diverse neuronal populations that project to multiple pain‐processing brain regions.^[^ ^16^ ^]^ Despite the lungs representing the largest innervated surface area—primarily via vagal fibers that relay peripheral signals to the CNS,^[^ ^17^ ^]^ the heterogeneity and functional roles of pulmonary sensory neurons remain poorly understood. Recent studies identified two distinct vagal afferent subtypes (*Npy2r* and *P2ry1* neurons) that differentially regulate respiration and target the nucleus tractus solitarius (NTS), a key brainstem hub for sensory integration.^[^ ^18^ ^]^ Precise characterization of the vagal sensory subtypes and body–brain circuits mediating MSC‐induced analgesia could unlock novel, targeted therapeutic strategies for pain management.

Our study identifies a novel lung‐to‐brain analgesic pathway mediated by *Npy2r* vagal sensory neurons in MSC‐induced analgesia. Intravenously delivered MSCs activate lung‐innervating *Npy2r* neurons, which project to the NTS and subsequently drive neuronal activation in the ventrolateral periaqueductal gray (vlPAG). Chemogenetic stimulation of these neurons replicated MSC‐induced analgesia in SNI mice. Mechanistically, MSC‐derived ATP activates *Npy2r* neurons via P2X2 receptors (P2rx2), and inhaled P2rx2 agonists similarly alleviated neuropathic pain. These findings establish the *Npy2r* sensory neuron‐mediated lung–brain axis as critical for MSC‐induced analgesia.

## 2 Results

### 2.1 MSC Alleviate Nerve Injury‐Induced Pain via vlPAG Neurons

**Figure 1:**
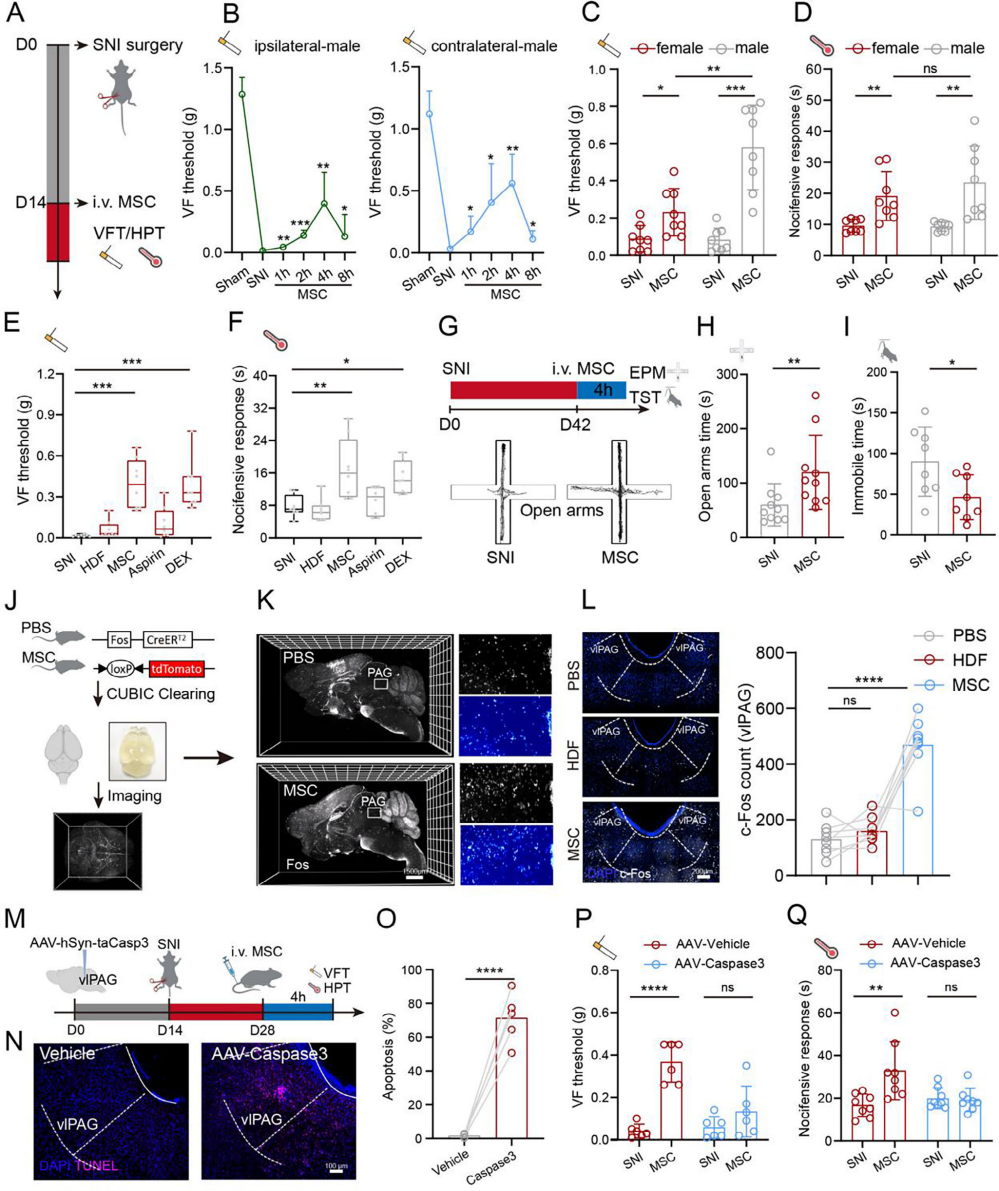
MSC alleviate nerve injury‐induced pain via vlPAG neurons. A) Schematic diagram for MSC therapy in SNI mice. von Frey test (VFT), Hot plate test (HPT). B) Paw withdrawal threshold of the ipsilateral paw (left) and contralateral paw (right) in male mice assessed by the von Frey test (VFT) at different time points after MSC injection (*n* = 5 mice). C) Mechanical nociceptive sensitivity of male (*n* = 8 mice) and female (*n* = 8 mice) SNI mice 4 h after MSC injection. Two‐tailed *t*‐test. D) Thermal nociceptive sensitivity of male (*n* = 8 mice) and female (*n* = 8 mice) SNI mice 4 h after MSC injection. Two‐tailed *t*‐test. E) Mechanical nociceptive sensitivity of male (*n* = 8 mice) SNI mice after 4 h of MSC injection or different drug delivery. Kruskal–Wallis test. DEX, Dexmedetomidine. F) Thermal nociceptive sensitivity of male (*n* = 8 mice) SNI mice after 4 h of MSC injection or different drug delivery. One‐way ANOVA. G) Upper: Schematic diagram for behavioral tests. Lower: Representative elevated plus maze (EPM) traces. H) Statistical analysis of the open arm time in the EPM (*n* = 10 mice). Mann–Whitney test. I) Statistical analysis of the immobile time in the TST (*n* = 8 mice). Two‐tailed *t*‐test. J) Schema for whole‐brain activity imaging in response to MSC injection. K) Whole‐brain imaging results showing the Fos expression from three independent experiments. Scale bar, 1500 µm. L) Representative immunofluorescence staining images and the statistical analysis showing the vlPAG c‐Fos expression (*n* = 8 mice). Scale bar, 200 µm. One‐way ANOVA. M) Schematic diagram showing the experimental procedures for vlPAG neuron elimination before SNI surgery. N) Representative images showing the apoptosis of vlPAG neurons induced by AAV injection to overexpress Caspase3. Scale bar, 100 µm. O) Quantification showing the apoptosis of vlPAG neurons (*n* = 5 mice). Two‐tailed *t*‐test. P) Paw withdrawal threshold assessed by the VFT after 4 h of MSC injection in mice with (AAV‐Caspase3) or without (AAV‐Vehicle) vlPAG neuron elimination (*n* = 6 mice). Two‐tailed *t*‐test. Q) Paw withdrawal latency assessed by the HPT after 4 h of MSC injection in mice with or without vlPAG neuron elimination (*n* = 8 mice). Mann–Whitney test. Illustrations created with BioRender.com. ^*^ *p* < 0.05, ^**^ *p* < 0.01, ^***^ *p* < 0.001, ^****^ *p* < 0.0001, ns‐no significant difference. Error bars indicate the SD. See also Figures S1 and S2 (Supporting Information).

We used a spared nerve injury (SNI) mouse model of chronic pain to investigate the impact of MSC on analgesia, and intravenously administered a total of 1 × 10^6^ bone marrow‐derived MSC to the mice two weeks after SNI as outlined in **Figure** **1**A. Compared with the sham treatment, the mechanical pain threshold decreased on the ipsilateral and contralateral paw of SNI male mice, while MSC improved the SNI‐induced allodynia bilaterally 1 h after administration and peaked at 4 h as measured by the von Frey test (VFT) (Figure 1B). Interestingly, we found that MSC treatment exerted a better improvement in mechanical allodynia in male mice than female mice in the VFT (*p* = 0.0046, Two‐tailed *t*‐test), while the results of the hot plate test (HPT) showed similar effects in both genders of mice 4 h after MSC infusion (Figure 1C,D). To assess the efficacy of MSC compared with analgesics, we selected aspirin, a non‐steroidal anti‐inflammatory drug, and dexmedetomidine (DEX), a highly selective α2 adrenergic receptor agonist, for the analgesic performance evaluation of MSC. To rule out the general effects of cell injection, we also used the human dermal fibroblasts (HDF) as a cell control. Our data showed that HDF had little influence on analgesia as measured by VFT and HPT, while MSC exhibited a significant analgesic effect (*p* = 0.0002, Kruskal–Wallis test), similar to DEX, and better than aspirin in the SNI male mice, after 4 h of drug delivery (Figure 1E,F). To evaluate the long‐term efficacy of MSC, we measured mechanical and thermal pain thresholds on days 1, 3, 7, and 14 after infusion (Figure S1A–C, Supporting Information). The analgesic effect peaked at day 1, remained significant on day 3 (*p* = 0.017, Two‐tailed *t*‐test), and disappeared by day 7. Consistently, motor coordination assessed by the rotarod test showed no impairment, indicating that MSC treatment did not cause adverse motor effects (Figure S1D, Supporting Information). We further tested whether MSC‐derived extracellular vesicles (MSC‐EV) could mimic the analgesic effects of MSC (Figure S1E–G, Supporting Information). Both MSC and MSC‐EV increased mechanical (Figure S1H, Supporting Information) and thermal thresholds (Figure S1I, Supporting Information), although the effects of MSC‐EV were less pronounced. These results suggest that MSC‐EV may offer a therapeutic option for neuropathic pain, albeit with reduced efficacy compared to whole‐cell MSC therapy. To broaden the scope, we tested MSC in other pain models. In a paclitaxel‐induced chemotherapy‐induced peripheral neuropathy (CIPN) model (Figure S1J, Supporting Information), MSC significantly attenuated mechanical (*p* = 0.0205, one‐way ANOVA, Figure S1K, Supporting Information) and thermal allodynia (*p* = 0.0024, one‐way ANOVA, Figure S1L, Supporting Information). Similarly, MSC reduced pain behaviors in the complete Freund's adjuvant (CFA)‐induced inflammatory pain model (Figure S1M–O, Supporting Information). Collectively, these findings indicate that MSC exhibit analgesic effects in diverse preclinical pain models.

Neuropathic pain has been reported to induce anxiety‐like and depressive symptoms in the SNI mouse model,^[^ ^19^ ^]^ we also observed that MSC infusion improved SNI‐induced anxiety‐like and depressive behaviors in the elevated plus maze (EPM) and the tail‐suspension test (TST) (Figure 1G–I). Considering the immunomodulatory function of MSC, we detected the serum IL‐6 levels in acute (24 h) and chronic (2 weeks) SNI mice 4 h after MSC injection. As shown in Figure S2A, the acute phase induced an increase in IL‐6 level, which could be alleviated by MSC injection. In contrast, MSC did not show notable anti‐inflammatory effects in the mice with chronic pain, and this study mainly focused on the therapeutic effect of MSC on the chronic phase of SNI mice. Overall, MSC exhibit similar pain‐reducing effects to dexmedetomidine in chronic pain mice, perhaps not through their anti‐inflammatory effects.

To investigate the reactivity of the brain that respond to MSC, we used the genetic mice (*Fos^CreERT2^; Loxp‐tdTomato*) and the whole‐brain clearing technology to identify sites of immediate‐early gene Fos expression after MSC injection (Figure 1J), and found that notable neural activation in the MSC injection condition versus PBS was the ventral lateral parts of periaqueductal gray area (vlPAG) (Figure 1K), hippocampus, ventral posterolateral (VPL) and ventral posteromedial (VPM) thalamus, and anterior cingulate cortex (ACC), etc. (Figure S2B, Supporting Information). Among these, the vlPAG is associated with the powerful pain control system existing in the brain.^[^ ^20^ ^]^ We confirmed the phenomenon with the immunofluorescence analysis that the MSC‐induced increased c‐Fos expressions in vlPAG (*p* < 0.0001, one‐way ANOVA, Figure 1L). To further determine whether vlPAG neurons were activated after MSC administration, we assessed the real‐time activity of vlPAG neurons using fiber photometry (Figure S2C,D, Supporting Information) and found MSC infusion could enhance average calcium signals in vlPAG (Figure S2E, Supporting Information). To find the causal connection of vlPAG neurons in analgesia of MSC therapy, we ablated vlPAG neurons by AAV‐hSyn‐taCasp3 injection (Figure 1M) to observe the analgesia after MSC injection, and the apoptosis in vlPAG region was determined via TUNEL assay (Figure 1N,O). As expected, the elimination of vlPAG neurons abolished the analgesic effects of MSC in SNI mice as measured by paw withdrawal threshold in the VFT and paw withdrawal latency in the HPT (Figure 1P,Q). To verify the neural connection of vlPAG to the peripheral paw, we mapped the neural circuit with recombinant pseudorabies virus (PRV‐EGFP), which infected from the hindpaw to the spinal cord, rostral ventromedial medulla (RVM), and vlPAG (Figure S2F, Supporting Information). In conclusion, these results suggest that MSC exert analgesic effects in SNI mice by vlPAG neurons.

### 2.2 MSC Regulate vlPAG Neurons via the Pulmonary Vagal→NTS→vlPAG Pathway

**Figure 2:**
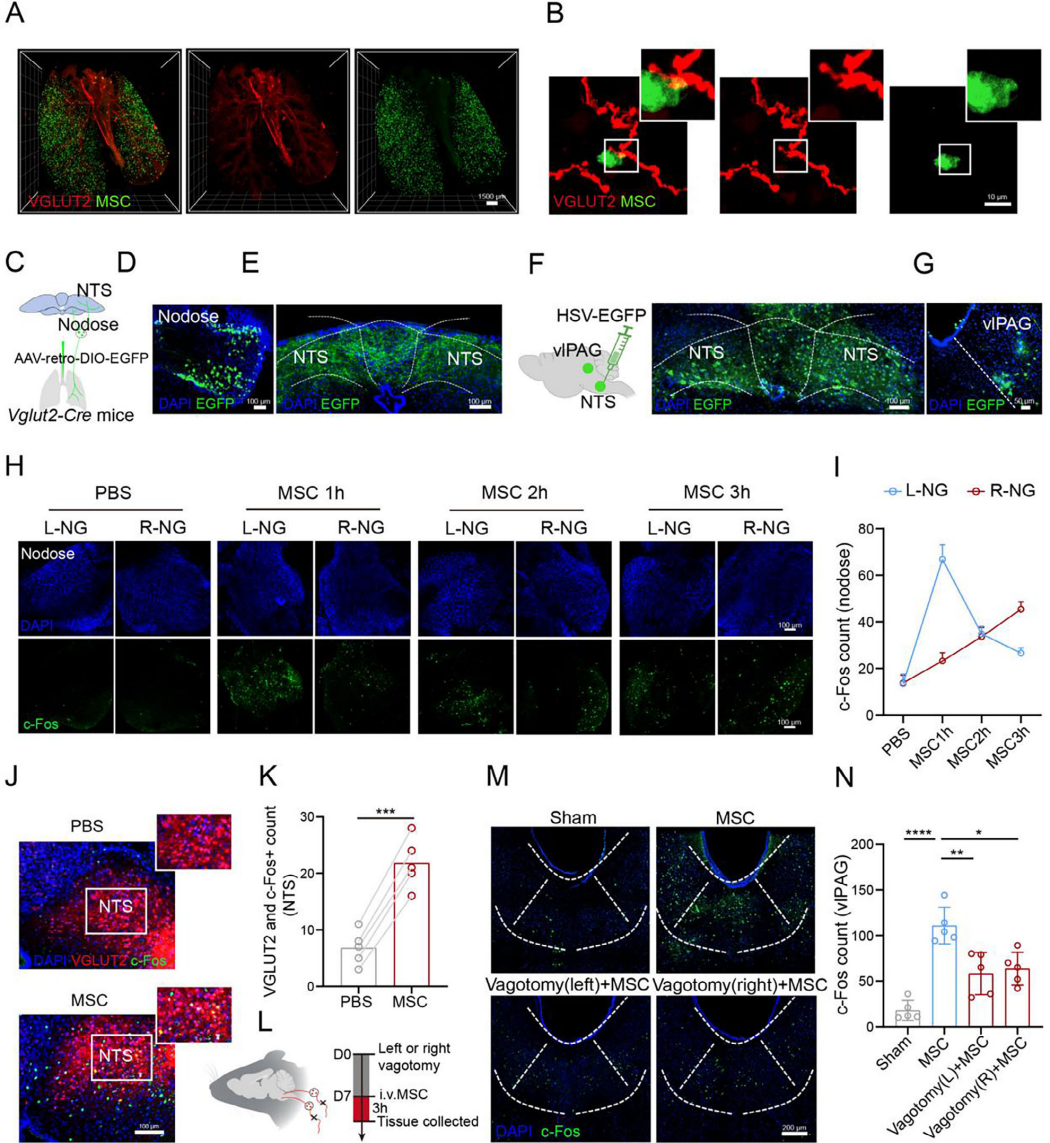
MSC regulate vlPAG neurons via the pulmonary vagal→NTS→vlPAG pathway. A) Representative 3D reconstructed light sheet images of pulmonary sensory nerve fibers (VGLUT2, red) and MSC (GFP, green) in the whole lung. Scale bar, 1500 µm. B) Representative immunofluorescence staining images from three independent experiments showing the colocalization between GFP‐MSC and pulmonary nerves (VGLUT2). Scale bar, 10 µm. C) Schematic of neural tracing of lung‐innervating sensory neurons by injecting the AAV‐retro‐DIO‐EGFP into the lungs of *VGLUT2‐Cre* mice. D) GFP‐labeled neurons in a nodose ganglion from three independent experiments. Scale bar, 100 µm. E) Fiber distribution of lung‐innervating sensory neurons in the NTS from three independent experiments. Scale bar, 100 µm. NTS, nucleus of the solitary tract. F) The polysynaptic herpes simplex virus, HSV‐GFP, was injected bilaterally into the NTS for anterograde tracing. Scale bar, 100 µm. G) Representative images from three independent experiments showing the HSV‐GFP‐infected neurons within the vlPAG. Scale bar, 50 µm. H) Time course study for investigating c‐Fos activation in the left (L) and right (R) nodose ganglia for 1, 2, and 3 h following MSC injection. Scale bar, 100 µm. I) The quantification of c‐Fos expression in the nodose ganglia (*n* = 5 mice). J,K) Representative images (J) and quantification (K) of VGLUT2 and c‐Fos colocalization in the NTS (*n* = 5 mice). Scale bar, 100 µm. Two‐tailed *t*‐test. L) The experimental procedures for vagotomy. M) Representative images showing the vlPAG c‐Fos expression. Scale bar, 200 µm. N) Statistical analysis of the vlPAG c‐Fos expression (*n* = 8 mice). One‐way ANOVA. Illustrations created with BioRender.com. ^*^ *p* < 0.05, ^**^ *p* < 0.01, ^***^ *p* < 0.001, ^****^ *p* < 0.0001, ns‐no significant difference. Error bars indicate the SD. See also Figure S3 (Supporting Information).

Intravenously infused MSC are mainly distributed in the lungs, where primarily innervated by a branch of the vagal sensory fibers.^[^ ^21^ ^]^ GFP‐labelled MSC were delivered intravenously to the *VGLUT2‐Cre; Loxp‐tdTomato* genetic mice, and we used tissue clearing and imaging technology to observe the whole lungs. Our results showed that the majority of VGLUT2 fibers run along the major airways beneath and parallel to the smooth muscle layer (Figure S3A, Supporting Information), and infused GFP‐MSC were in close proximity to vagal sensory nerves in the lungs (**Figure** **2**A,B). Additionally, GFP+ cells in the lung expressed the MSC‐specific markers CD105 and CD90, indicating that the infused cells retained MSC characteristics within the 3‐day therapeutic window (Figure S3B, Supporting Information). To further evaluate viability and functionality, we isolated GFP+ cells by FACS at day 3 (Figure S3C, Supporting Information) and assessed cell survival using LIVE/DEAD staining. Over half of the isolated cells were viable (Figure S3D, Supporting Information). Transcriptomic analysis, based on our previous dataset (GSE246290), further confirmed the expression of MSC markers CD73, CD90, and CD105, and the absence of hematopoietic lineage markers CD3, CD19, CD34 in GFP‐MSC isolated from lung homogenates three days post‐infusion (Figure S3E, Supporting Information). Critically, both cultured and lung‐isolated GFP‐MSC showed minimal HLA‐DR expression (Figure S3F, Supporting Information). These results support the viability and functional activity of infused MSC during this effective window for MSC treatment.

Next, we hypothesized that intravenous MSC regulated vlPAG via pulmonary vagal afferents. To map the circuit, we injected AAV‐retro‐DIO‐EGFP into the lungs of *VGLUT2‐Cre* mice to mark the vagus nerves innervating lungs (Figure 2C) and observed remarkable signals in nodose ganglia and nucleus tractus solitarius (NTS) (Figure 2D,E). Then, we injected anterograde tracing virus HSV‐EGFP into NTS (Figure 2F), and the infection extended to vlPAG targets (Figure 2G). Furthermore, we noticed obvious c‐Fos signals of nodose ganglia on both sides after MSC injection in a time‐dependent way and found that the left nodose ganglia appeared to respond earlier to MSC infusion (Figure 2H,I). Additionally, we also recorded the calcium signal of nodose ganglia in the *VGLUT‐Cre; Loxp‐GCaMP6* mice after MSC or HDF infusion (Figure S3G, Supporting Information). While HDF injection only increased the calcium signaling in a small group of vagal sensory neurons (5/16 neurons), MSC injection evoked calcium transients in almost all recorded vagal sensory neurons (20 neurons). Within the imaged nodose ganglia, there are differences in the response time and intensity of various sensory neurons, indicating that MSC‐responding vagal sensory neurons are heterogeneous (Figure S3H, Supporting Information). Then, we also collected the nodose ganglia after calcium imaging and we observed that MSC injection group showed a higher c‐Fos expression in the VGLUT2‐GCaMP6 positive sensory neurons, compared with HDF injection (Figure S3I, Supporting Information). Furthermore, MSC injection induced the neuron activation in NTS (Figure S3J, Supporting Information) and the increase in the colocalization of c‐Fos and VGLUT2, suggesting MSC‐induced activation of glutamatergic neurons in the NTS (*p* = 0.0003, Two‐tailed *t*‐test, Figure 2J,K). To further test whether MSC induced vlPAG activation via vagal signaling, we performed vagotomy before MSC injection (Figure 2L). Vagotomy partially inhibited the activation of NTS neurons (Figure S3K,L, Supporting Information) and vlPAG neurons (Figure 2M,N) induced by MSC. In summary, these results reveal that MSC might relay signals to the vlPAG via the pulmonary vagal–brain axis.

### 2.3 Lung‐Innervating *Npy2r* Sensory Neurons Exert Analgesic Effects

**Figure 3:**
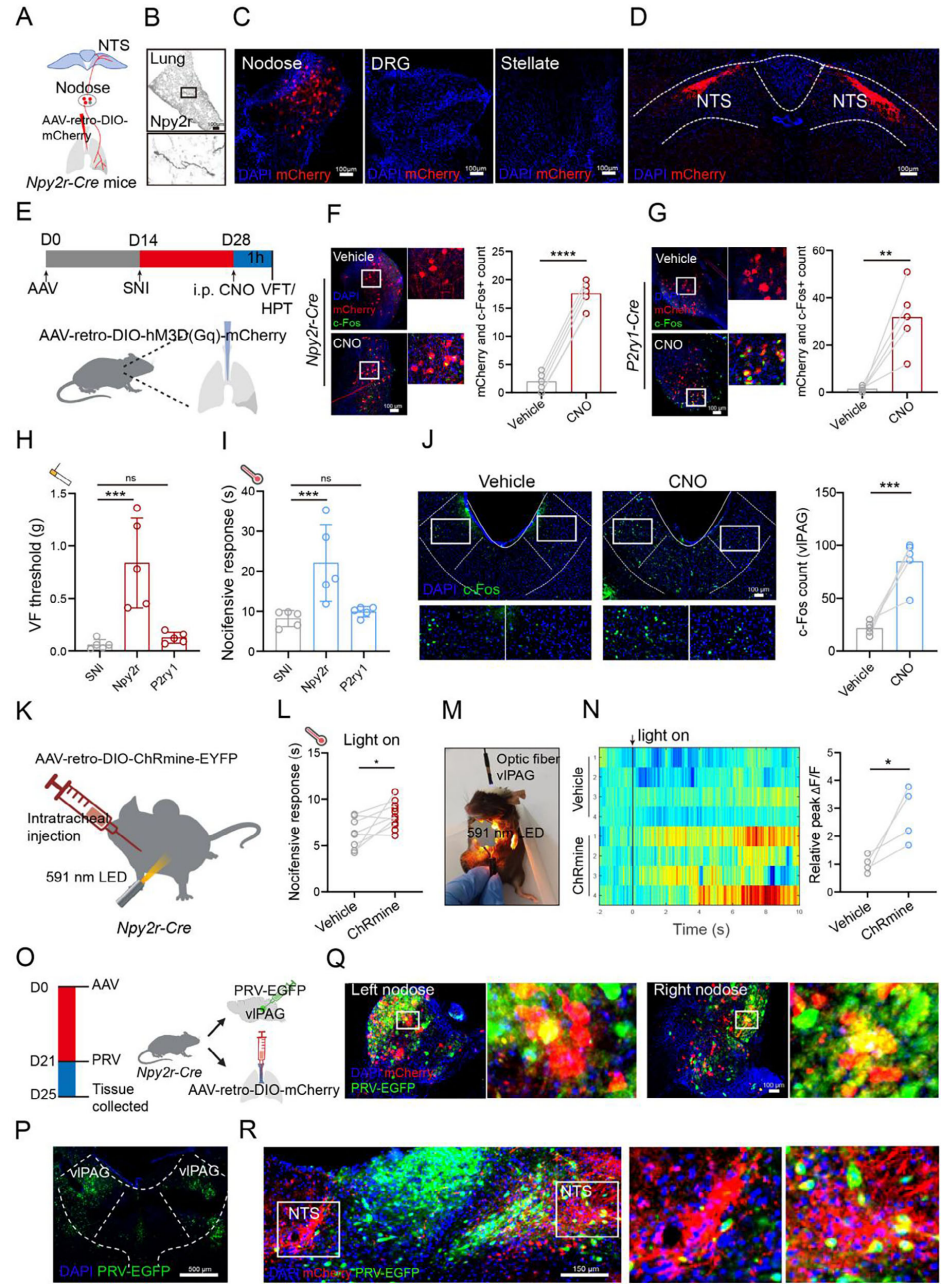
Lung‐innervating *Npy2r* sensory neurons exert analgesic effects. A) Schematic of neural tracing of *Npy2r* sensory neurons innervating lungs. B) Representative images showing the terminals of lung‐innervating *Npy2r*‐expressing vagal sensory neurons. Scale bar, 100 µm. C) mCherry‐labeled neurons in a nodose ganglion, dorsal root ganglion (DRG), and stellate ganglion of a *Npy2r‐Cre* mouse from three independent experiments. Scale bar, 100 µm. D) Fiber distribution of *Npy2r* sensory neurons innervating lungs in the NTS from three independent experiments. Scale bar, 100 µm. E) Schematic diagram showing the experimental procedures used for hM3Dq‐mediated lung‐innervating *Npy2r* or *P2ry1* neuron activation. AAV‐retro‐DIO‐hM3Dq‐mCherry was injected into the lungs through the trachea. F) Representative images and the quantification showing the colocalization between mCherry (hM3Dq‐mediated lung‐innervating *Npy2r* sensory neuron) and c‐Fos in the nodose ganglia (*n* = 5 mice). Scale bar, 100 µm. Two‐tailed *t*‐test. G) Representative images and the quantification showing the colocalization between mCherry (hM3Dq‐mediated lung‐innervating *P2ry1* sensory neuron) and c‐Fos in the nodose ganglia (*n* = 5 mice). Scale bar, 100 µm. Two‐tailed *t*‐test. H) Paw withdrawal threshold assessed by the VFT in different experimental groups (*n* = 5 mice). One‐way ANOVA. I) Paw withdrawal latency assessed by the HPT in different experimental groups (*n* = 5 mice). One‐way ANOVA. J) Representative images and the quantification showing that the hM3Dq‐mediated lung‐innervating *Npy2r* sensory neuron activation induced the increase in the vlPAG c‐Fos expression (*n* = 5 mice). Scale bar, 100 µm. Two‐tailed *t*‐test. K) Schematic diagram of the ChRmine optogenetics stimulation. L) The ChRmine optogenetics‐mediated lung‐innervating *Npy2r* sensory neuron activation improved paw withdrawal latency assessed by the HPT (Vehicle, *n* = 8 mice; ChRmine, *n* = 12 mice). Two‐tailed *t*‐test. M) The optic fiber recordings in vlPAG upon ChRmine‐mediated stimulation of *Npy2r* sensory neurons innervating lungs. N) Left: Heatmaps of spontaneous calcium signals in the vehicle and ChRmine mice. Right: Statistical analysis showed relative peak fluorescence intensity changes (ΔF/F) in vlPAG (*n* = 4 mice). Two‐tailed *t*‐test. O) Schematic of two‐virus neural tracing by using AAV‐retro‐DIO‐mCherry and PRV‐EGFP. P) The PRV‐EGFP was injected bilaterally into the vlPAG for retrograde tracing. Scale bar, 500 µm. Q,R) The merge of infected mCherry and GFP signal in nodose ganglia (Q) and NTS (R) from three independent experiments. Scale bar, 100 µm. Illustrations created with BioRender.com. ^*^ *p* < 0.05, ^**^ *p* < 0.01, ^***^ *p* < 0.001, ^****^ *p* < 0.0001, ns‐no significant difference. Error bars indicate the SD. See also Figures S4–S6 (Supporting Information).

The afferent sensory ganglia likely contain a diversity of molecularly distinct neuron types with different anatomical projections and functions. Among these, sensory neurons of the vagus nerve are the major source of nerve fibers that innervate the lung and airways.^[^ ^22^ ^]^ We next sought to identify the subtypes of pulmonary vagal sensory neurons driving analgesia. A previous study uncovered that *P2ry1* and *Npy2r* vagal sensory neurons may relate to distinct lung‐to‐brain connectivity.^[^ ^18^ ^]^ *P2ry1* neurons are mostly fast‐conducting A fibers conveying to lateral NTS, while *Npy2r* neurons target a medial posterior region of the NTS that receives pulmonary C fiber input. Consistently, we reanalyzed single‐cell RNA sequencing (scRNA‐seq) data of the nodose ganglia (GSE145216), *Npy2r* vagal sensory neurons formed a distinct genetic cluster from *P2ry1* subpopulation (Figure S4A,B, Supporting Information). To map the lung‐innervating *Npy2r* and *P2ry1* sensory neurons relatively, we first injected AAV‐retro‐DIO‐mCherry into the lungs of *Npy2r‐Cre* mice (**Figure** **3**A,B). For lung innervating sensory neurons, we examined nodose ganglia, thoracic dorsal root ganglia (DRG), and stellate ganglia. After infection, nodose ganglia, instead of the DRG and stellate ganglia, showed infection signals (Figure 3C), and we also observed the signals in NTS (Figure 3D). Next, we injected AAV‐retro‐P2ry1‐Cre and AAV‐retro‐DIO‐mCherry into the lungs of wild‐type mice to observe lung‐innervating *P2ry1* vagal sensory neurons (Figure S4C, Supporting Information), and the signal also showed in nodose ganglia and the NTS (Figure S4D, Supporting Information). Additionally, we observed different afferent terminal morphology between lung‐innervating *Npy2r* and *P2ry1* sensory neurons (Figure S4E, Supporting Information). Next, to determine the role of lung‐innervating *Npy2r* or *P2ry1* sensory neurons in analgesia, we used chemogenetics to activate lung‐innervating *Npy2r* or *P2ry1* sensory neurons relatively and assessed analgesic effects. According to a previous study, intratracheal instillation of retrograde AAV‐retro virus can label pulmonary sensory nerves, and most sensory neurons were labeled in the nodose ganglia.^[^ ^23^ ^]^ Therefore, for the activation of vagal sensory *Npy2r* neurons that innervate the lung, AAV‐retro‐DIO‐hM3Dq‐mCherry was injected into the lungs through the trachea in *Npy2r‐Cre* mice (Figure 3E), and the positive signals of infection mainly appeared in pulmonary nerves rather than other organs (Figure S5A, Supporting Information). Then, we observed the increase in c‐Fos expression in the mCherry+ neurons of nodose ganglia after CNO injection (Figure 3F). Moreover, for the activation of lung‐innervating *P2ry1* sensory neurons, we injected a mixture of two kinds of AAV (AAV‐retro‐DIO‐hM3Dq‐mCherry and AAV‐retro‐P2ry1‐Cre) into the lungs through the trachea in wild‐type mice, and we also observed the increase in c‐Fos expression in the mCherry+ neurons of nodose ganglia after CNO injection (Figure 3G). *Npy2r* sensory neuron activation, instead of *P2ry1* sensory neuron, improved SNI‐induced mechanical allodynia (*p* = 0.0010, one‐way ANOVA) and thermal hyperalgesia (*p* = 0.0059, one‐way ANOVA) after activation (Figure 3H,I), which is CNO dose‐dependent (Figure S5B, Supporting Information). In addition, we also observed the facial expressions of mice in different groups. Compared with mice in the SNI group, mice with pulmonary *Npy2r* sensory neuron activation did not show excessive suffering (Figure S5C, Supporting Information). Additionally, hM3Dq‐mediated lung‐innervating *Npy2r* sensory neuron activation induced an increase in c‐Fos expression of vlPAG (*p* = 0.0002, Two‐tailed *t*‐test, Figure 3J). Similarly, we expressed the highly sensitive and red‐shifted pump‐like channelrhodopsin (ChRmine) in lung‐innervating *Npy2r* vagal sensory neurons by injecting AAV‐retro‐DIO‐ChRmine‐EYFP into the lungs of *Npy2r‐Cre* mice (Figure 3K). After shining 591 nm LED light on the chest of mice for 1 min, SNI mice showed improved thermal hyperalgesia in the HPT (*p* = 0.0142, Two‐tailed *t*‐test, Figure 3L). In addition, we assessed the real‐time activity of vlPAG neurons using fiber photometry (Figure S5D–F, Supporting Information) and detected an increase in average calcium signals upon ChRmine‐mediated stimulation of lung‐innervating *Npy2r* vagal sensory neurons (Figure 3M,N, Figure S5G, Supporting Information). Consistently, we observed that the firing rate of vlPAG neurons was increased after light on the chest of mice (Figure S5H,I, Supporting Information). These results demonstrate that selective activation of lung‐innervating *Npy2r* sensory neurons produced analgesia in SNI mice, establishing a potential lung‐to‐brain circuit for pain modulation.

We questioned whether lung‐innervating *Npy2r* vagal sensory neurons could also project to vlPAG. To determine this, we performed a two‐virus injection strategy as outlined in Figure 3O. Specifically, we injected AAV‐retro‐DIO‐mCherry into the lungs of *Npy2r‐Cre* mice on day 0, and PRV‐EGFP into vlPAG on day 21 (Figure 3P). After 96 h of infection, we observed the merge of red fluorescence signal and green fluorescence signal in nodose ganglia and NTS, indicating the potential projection of *Npy2r* vagal sensory neurons innervating lungs to vlPAG (Figure 3Q,R). In addition, we also injected fast blue and PRV‐EGFP into a wild‐type mouse, which showed similar tracing results (Figure S6A–C, Supporting Information). Taken together, our results suggest that *Npy2r* sensory neurons drive analgesia via the lung‐to‐brain pathway.

### 2.4 MSC Activate *Npy2r* Sensory Neurons for Analgesia

**Figure 4:**
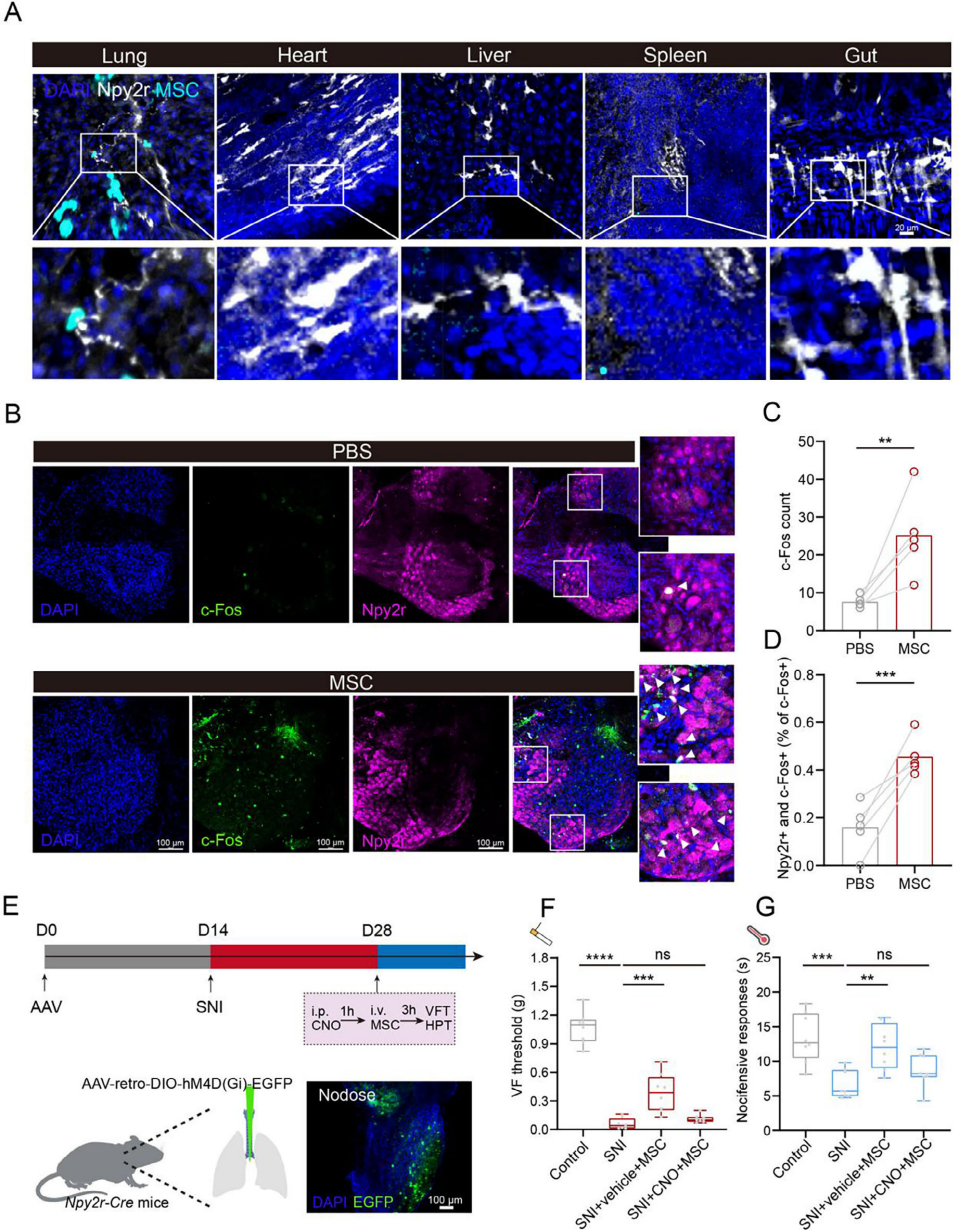
MSC activate *Npy2r* sensory neurons for analgesia. A) Representative images from three independent experiments showing GFP‐MSC and *Npy2r* distribution in different organs. Scale bar, 20 µm. B—D) Representative images (B) and quantification (C and D) of *Npy2r* and c‐Fos colocalization in nodose ganglia after MSC injection (*n* = 5 mice). Scale bar, 100 µm. Two‐tailed *t*‐test. E) Upper: Schematic diagram for hM4Di‐mediated lung‐innervating *Npy2r* neuron silencing. Lower: AAV‐retro‐DIO‐hM4Di‐EGFP was injected into the lungs of *Npy2r‐Cre* mice through the trachea. The infection signal of AAV in the nodose ganglia of *Npy2r‐Cre* mice. Scale bar, 100 µm. F) Paw withdrawal threshold assessed by the VFT in different experimental groups (*n* = 8 mice). One‐way ANOVA. G) Paw withdrawal latency assessed by the HPT in different experimental groups (*n* = 8 mice). One‐way ANOVA. Illustrations created with BioRender.com. ^*^ *p* < 0.05, ^**^ *p* < 0.01, ^***^ *p* < 0.001, ^****^ *p* < 0.0001, ns‐no significant difference. Error bars indicate the SD. See also Figure S7 (Supporting Information).

As lung‐innervating *Npy2r* vagal sensory neurons could project to vlPAG, we questioned whether the MSC play an analgesic role through pulmonary *Npy2r* sensory neurons. We first observed the distribution of infused GFP‐MSC and *Npy2r* sensory nerves in different organs, including the lung, heart, liver, spleen, and gut. The colocalization between GFP‐MSC and *Npy2r* sensory nerves was only shown in the lungs, indicating the potential crosstalk between MSC and pulmonary *Npy2r* sensory nerves (**Figure** **4**A). Moreover, we also performed the co‐staining of neuronal marker (TUBB3) in the lungs of *Npy2r‐Cre; Loxp‐tdTomato* mice and *Npy2r* expressed in the pulmonary nerves (Figure S7A, Supporting Information). Furthermore, MSC induced an increase in the c‐Fos expression in *Npy2r* sensory neurons of nodose ganglia (Figure 4B–D), suggesting that MSC injection might induce the activation of *Npy2r* vagal sensory neurons in the nodose ganglia. To further confirm this hypothesis, AAV‐retro‐DIO‐hM4D(Gi)‐EGFP was injected into the lungs of *Npy2r‐Cre* mice to silence the *Npy2r* sensory neurons innervating lungs (Figure 4E). In the VFT, MSC improved the mechanical pain threshold of SNI mice in the vehicle group, while the hM4Di‐mediated silencing of *Npy2r* sensory neurons inhibited the analgesic effects of MSC (Figure 4F). In the HPT, the inhibition of *Npy2r* sensory neurons abolished MSC‐induced thermal hyperalgesia improvement in SNI mice (Figure 4G). Overall, these findings suggest that the activation of lung‐innervating *Npy2r*‐expressing sensory neurons contributes to the analgesic effects of MSC.

### 2.5 Increased Calcium Signaling in Lung‐Innervating *Npy2r* Sensory Neurons by Peripheral MSC

**Figure 5:**
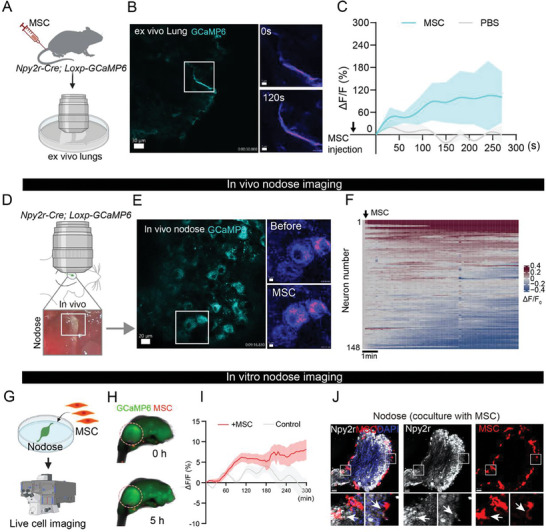
Increased calcium signaling in lung‐innervating *Npy2r* sensory neurons by peripheral MSC. A) Schematic diagram showing the ex vivo lung calcium imaging after MSC injection or PBS injection. B) Representative sequential images from three independent experiments displaying calcium signal response to infused MSC inside the ex‐vivo lungs of *Npy2r‐cre; Loxp‐GCaMP6* mice. Scale bar, 30 µm. C) Real‐time changes in fluorescence intensity were expressed as percentage changes over baseline (ΔF/F). *n* = 3 mice. D) Cartoon depicting in vivo vagal ganglion imaging after MSC injection. E) Representative sequential images from three independent experiments displaying increased calcium signal from *Npy2r* vagal neurons responding to MSC infusion inside the in vivo nodose ganglia of *Npy2r‐cre; Loxp‐GCaMP6* mice. Scale bar, 20 µm. F) Heatmap depicting vagal sensory neuron calcium responses (ΔF/F, 148 imaged neurons, 3 representative mice) to MSC injection. G) Schematic diagram for the in vitro nodose ganglia coculture with RFP‐MSC. H) Representative sequential images from three independent experiments displaying calcium signal response to cocultured RFP‐MSC inside the in vitro nodose ganglia. I) Real‐time changes in fluorescence intensity of in vitro nodose ganglia were expressed as percentage changes over baseline (ΔF/F). *n* = 3 nodose ganglia. J) Representative images showing the RFP‐MSC distribution and *Npy2r* expression in the cocultured nodose ganglia. Scale bar, 50 µm. Illustrations created with BioRender.com. See also Figure S8 (Supporting Information).

To investigate whether MSC infusion elicited responsiveness in pulmonary *Npy2r* sensory fibers, we crossed *Npy2r‐Cre* mice with *Loxp‐GCaMP6* mice to get *Npy2r‐Cre; Loxp‐GCaMP6* genetic mice, and MSC were delivered intravenously to the genetic mice. Then, we recorded the activity of *Npy2r* sensory fibers in the fresh ex vivo lungs (**Figure** **5**A). We found that pulmonary *Npy2r* sensory fibers were stimulated by MSC, as measured by GCaMP6 fluorescence intensity, compared with the PBS group (Figure 5B,C). Moreover, we also performed the co‐staining of neuronal marker (TUBB3) in the lungs of *Npy2r‐cre; Loxp‐GCaMP6* mice and *Npy2r‐GCaMP6* expressed in the pulmonary nerves (Figure S8A, Supporting Information). Next, we used in vivo calcium imaging within the vagal ganglia to investigate *Npy2r* vagal sensory neuron responses to MSC injection (Figure 5D; Figure S8B, Supporting Information). We imaged vagal ganglia while connections to the lungs were preserved. As expected, MSC evoked calcium transients in most observed *Npy2r* vagal sensory neurons (121/148 neurons) (Figure 5E,F). To specifically confirm the lung‐innervating *Npy2r*‐expressing vagal sensory neurons responding to MSC, we injected the AAV‐retro‐DIO‐GCaMP6 into the lungs of *Npy2r‐Cre* mice (Figure S8C, Supporting Information) and recorded calcium signals in the nodose ganglia after MSC injection (Figure S8D, Supporting Information). As expected, most lung‐innervating neurons (14/16 neurons) respond to MSC infusion (Figure S8E, Supporting Information). To further observe the effects of MSC on *Npy2r* vagal sensory neurons, we also collected nodose ganglia from *Npy2r‐Cre; Loxp‐GCaMP6* mice and cocultured them with RFP‐MSC for several hours (Figure 5G). Of note, MSC could gently and sustainably increase neuronal calcium responses for at least 5 h in vitro (Figure 5H,I). Then, the cocultured nodose ganglia were observed by immunofluorescence analysis. Interestingly, we observed that MSC were located close to the co‐cultured nodose ganglia (Figure 5J). Together, these findings indicate that intravenous MSC increased calcium signaling in *Npy2r* vagal sensory neurons.

### 2.6 MSC Activate *Npy2r* Sensory Neurons via ATP Signaling

**Figure 6:**
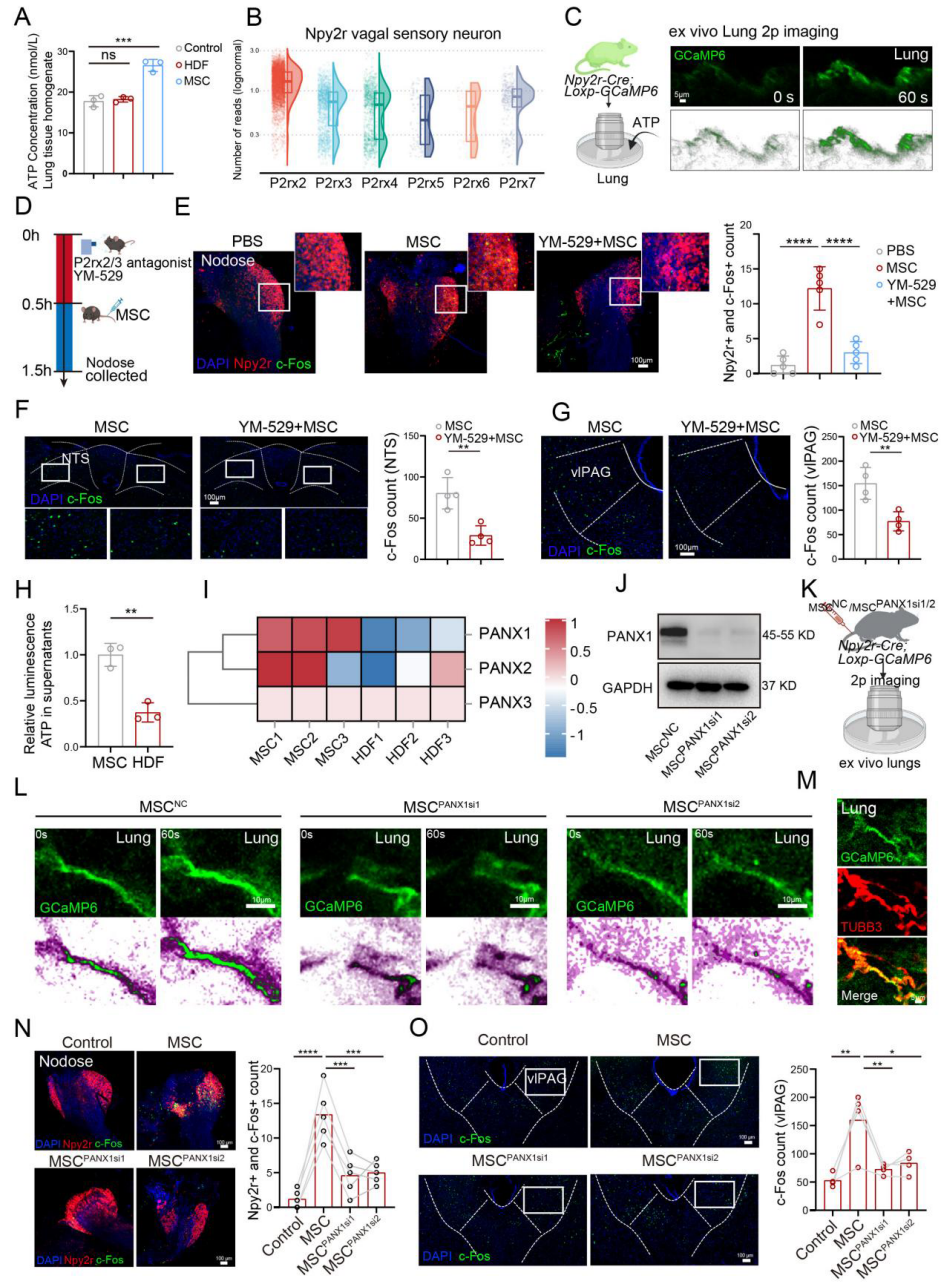
MSC activate *Npy2r* sensory neurons via ATP signaling. A) The concentration of ATP in lung tissue homogenates from different groups. *n* = 3 mice. One‐way ANOVA. B) Violin plots showing the expression of ligand‐gated purinergic receptors in *Npy2r* sensory neurons. C) Left: Schematic diagram for ex vivo lung calcium imaging. Right: Representative images displaying calcium signal response to ATP solution inside the ex‐vivo lungs of *Npy2r‐Cre; Loxp‐GCaMP6* mice. Scale bar, 5 µm. D) Schematic diagram showing the procedures used for MSC treatment and drug inhalation. E) Representative images and quantification of *Npy2r* and c‐Fos colocalization in nodose ganglia (*n* = 5 mice). Scale bar, 100 µm. One‐way ANOVA. F,G) Representative images and quantification of c‐Fos expression in NTS (F) and vlPAG (G) after YM‐529 treatment (*n* = 4 mice). Scale bar, 100 µm. Two‐tailed *t*‐test. H) Relative ATP level in supernatants of MSC and HDF. *n* = 3 biologically independent samples. Two‐tailed *t*‐test. I) Bulk RNA sequencing showing the expression of pannexin (PANX1‐3) in MSC and HDF. J) Western blotting confirming the siRNA‐mediated downregulation of PANX1 in MSC. K) Schematic diagram for ex vivo lung calcium imaging. L) Representative images displaying calcium signals inside the ex‐vivo lungs of *Npy2r‐Cre; Loxp‐GCaMP6* mice. Scale bar, 10 µm. M) Representative images showing the colocalization between *Npy2r*‐GCaMP6 and TUBB3 in the lungs. Scale bar, 50 µm. N) Representative images and quantification of *Npy2r* and c‐Fos colocalization in nodose ganglia (*n* = 5 mice). Scale bar, 100 µm. One‐way ANOVA. O) Representative images and quantification of c‐Fos expression in vlPAG (*n* = 4 mice). Scale bar, 100 µm. One‐way ANOVA. Illustrations created with BioRender.com. ^*^ *p* < 0.05, ^**^ *p* < 0.01, ^***^ *p* < 0.001, ^****^ *p* < 0.0001, ns‐no significant difference. Error bars indicate the SD. See also Figure S9 (Supporting Information).

Previous studies reported that extracellular ATP could stimulate vagal C fibers in the lung mediated by P2rx2/3 receptors located on vagal sensory nerve terminals.^[^ ^24^ ^]^ To further investigate how MSC interact with *Npy2r* sensory neurons, we first detected the ATP level of lung tissue homogenates after MSC and HDF injection. Compared with the HDF injection group, MSC injection increased the ATP level of lung tissue homogenates (*p* = 0.0003, one‐way ANOVA, **Figure** **6**A). Therefore, we hypothesized that MSC might activate pulmonary *Npy2r* sensory neurons by ATP signaling. To testify to this, we reanalyzed the published scRNA‐seq data (GSE145216) of the nodose ganglia, which suggested the higher expression of P2rx2 and P2rx3 in *Npy2r* sensory neurons compared with other ligand‐gated ion channel purinergic receptors (Figure 6B). In addition, we observed that fluorescence intensity was higher in pulmonary *Npy2r* sensory nerves of *Npy2r‐Cre; Loxp‐GCaMP6* genetic mice after adding ATP (Figure 6C). Furthermore, we used minodronic acid (synonyms: YM‐529), an antagonist of purinergic P2rx2/3 receptors, to confirm that MSC activated *Npy2r* vagal sensory neurons via P2rx2/3 receptor‐mediated ATP signaling (Figure 6D). The delivery of YM‐529 by nebulizer inhalation before MSC infusion was found to abrogate MSC‐mediated *Npy2r* neuron activation in nodose ganglia (Figure 6E). Additionally, the inhalation of YM‐529 also reduced the MSC‐induced c‐Fos signals in NTS (Figure 6F) and vlPAG (Figure 6G). Taken together, our results indicate that MSC activate *Npy2r* vagal sensory neurons to positively regulate CNS partially via ATP signaling.

ATP can be released from cells via vesicles or channel proteins, such as pannexin1 (PANX1).^[^ ^25^ ^]^ Accordingly, we performed quinacrine staining to detect ATP vesicle number by flow cytometry (Figure S9A, Supporting Information), and the results showed a bare difference in the ATP vesicle number of the MSC and HDF supernatants (Figure S9B, C, Supporting Information), while the ATP level in the MSC supernatant was higher than HDF (*p* = 0.0026, Two‐tailed t test, Figure 6H). Additionally, bulk RNA sequencing analysis suggested that the PANX1 expression in MSC was higher than HDF (Figure 6I). To evaluate whether MSC communicate with *Npy2r* neurons via PANX1‐regulated ATP release, we knocked down the expression of PANX1 in MSC (MSC^PANX1si1^ and MSC^PANX1si2^), as measured by qRT‐PCR, ATP concentration detection, and WB (Figure S9D, E, Supporting Information; Figure 6J). Next, we recorded the calcium activity of *Npy2r* sensory fibers in the fresh ex vivo lungs of *Npy2r‐Cre; Loxp‐GCaMP6* mice after MSC^NC^ injection or MSC^PANX1si1/2^ injection (Figure 6K). Compared with the MSC^NC^ group, MSC^PANX1si1^ and MSC^PANX1si2^ infusion barely elicited responsiveness in pulmonary *Npy2r* sensory fibers (Figure 6L; Figure S9F, Supporting Information). Moreover, the co‐staining of neuronal marker (TUBB3) in the lungs of *Npy2r‐cre; Loxp‐GCaMP6* mice confirmed that *Npy2r‐GCaMP6* expressed in the pulmonary nerves (Figure 6M). Furthermore, results of immunofluorescence analysis demonstrated that MSC with PANX1 knockdown scarcely activated *Npy2r* vagal sensory neurons in the nodose ganglia, compared with MSC^NC^ (Figure 6N). Consistently, the expression of c‐Fos in vlPAG exhibited a similar trend (Figure 6O). To establish the in vivo role of MSC‐derived ATP in activating *Npy2r* sensory neurons, we recorded calcium signals in *Npy2r* vagal neurons in the nodose ganglia. MSC infusion evoked calcium transients, which were suppressed by intratracheal administration of the purinergic receptor antagonist, YM‐529. Similarly, PANX1 knockdown in MSC abolished neuronal activation (Figure S9G,H, Supporting Information). Behaviorally, both YM‐529 treatment and PANX1‐deficient MSC reduced MSC‐induced analgesia in the VFT and HPT (Figure S9I–L, Supporting Information). These results indicate that MSC activate *Npy2r* sensory neurons and exert analgesic effects via ATP signaling.

### 2.7 Adenosine 5′‐(3‐Thiotriphosphate) Tetralithium Salt (ATPγS, a P2rx2 Agonist) Activating *Npy2r* Sensory Neurons Reduces Pain Response

**Figure 7:**
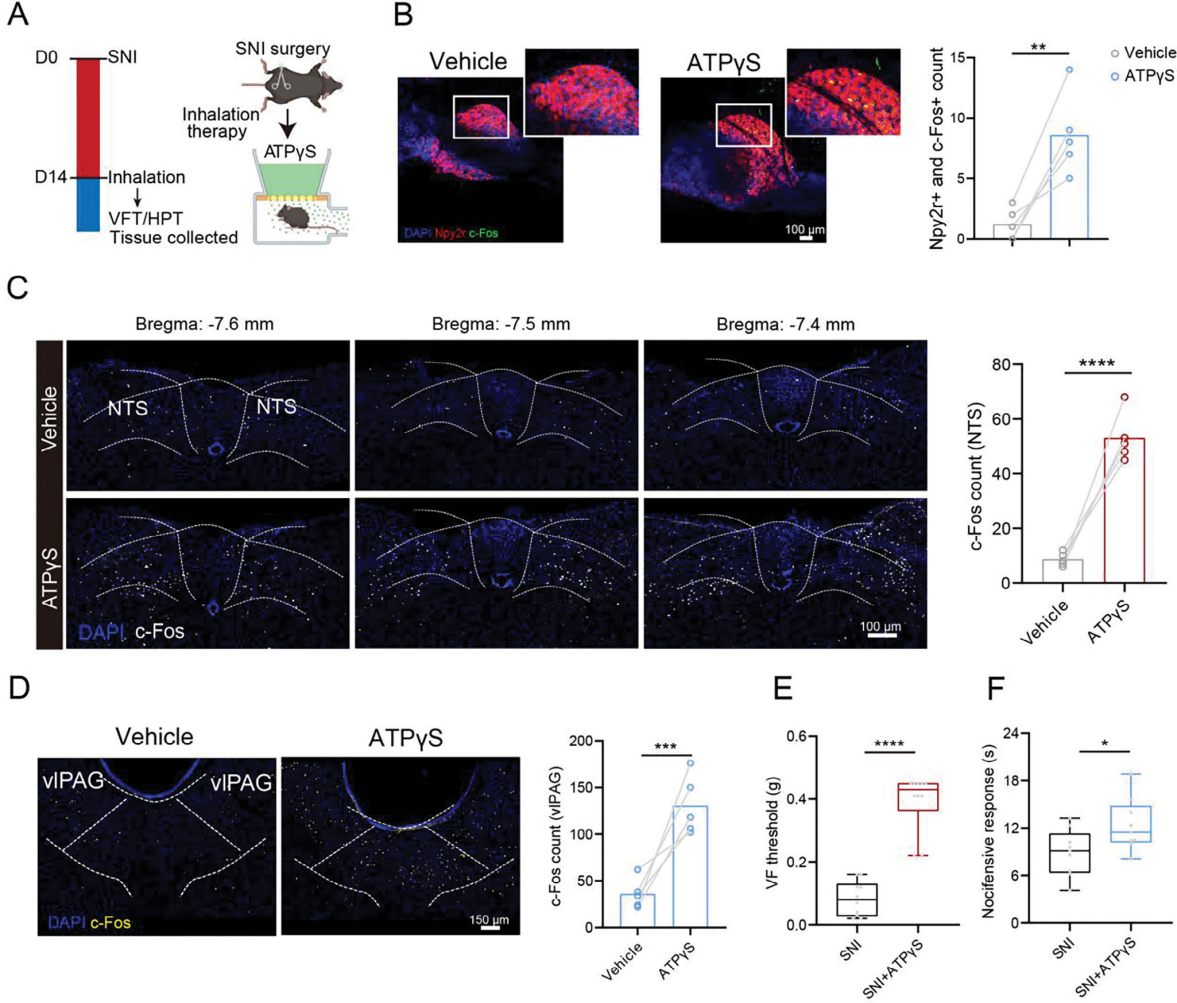
Adenosine 5′‐(3‐thiotriphosphate) tetralithium salt (ATPγS, a P2rx2 agonist) activating *Npy2r* sensory neurons reduces pain response. A) Schematic diagram showing the procedures for drug inhalation. B) Representative images and quantification of *Npy2r* and c‐Fos colocalization in nodose ganglia after Adenosine 5′‐(3‐thiotriphosphate) tetralithium salt (ATPγS, a P2rx2 agonist) inhalation (*n* = 5 mice). Scale bar, 100 µm. Two‐tailed *t*‐test. C) Representative images and quantification of c‐Fos expression in NTS after ATPγS inhalation (*n* = 5 mice). Scale bar, 100 µm. Two‐tailed *t*‐test. D) Representative images and quantification of c‐Fos expression in vlPAG after ATPγS inhalation (*n* = 5 mice). Scale bar, 100 µm. Two‐tailed *t*‐test. E) Paw withdrawal threshold assessed by the VFT after ATPγS inhalation (*n* = 10 mice). Mann Whitney test. F) Paw withdrawal latency assessed by the HPT after ATPγS inhalation (*n* = 9 mice). Two‐tailed *t*‐test. Illustrations created with BioRender.com. ^*^ *p* < 0.05, ^**^ *p* < 0.01, ^***^ *p* < 0.001, ^****^ *p* < 0.0001, ns‐no significant difference. Error bars indicate the SD. See also Figure S10 (Supporting Information).

To find a more convenient way to activate the pathway, we questioned whether direct inhalation of Adenosine 5′‐(3‐thiotriphosphate) tetralithium salt (ATPγS, a P2rx2 agonist), a P2rx2 agonist, could also activate *Npy2r* sensory neuron‐related lung‐to‐brain axis and produce a similar antinociceptive profile in SNI mice. As shown in **Figure** **7**A, SNI mice were placed in a confined space for aerosol inhalation of either PBS or ATPγS. First, we observed the activation of the *Npy2r* vagal sensory neurons in the nodose ganglia after inhalation (Figure 7B). Additionally, ATPγS inhalation also induced the activation of neurons in NTS (Figure 7C) and vlPAG (Figure 7D). More importantly, inhalation of ATPγS significantly improved SNI‐induced mechanical allodynia (*p* < 0.0001, Mann Whitney test) and thermal hyperalgesia (*p* = 0.0309, Two‐tailed *t*‐test) (Figure 7E,F). To further evaluate the efficacy and safety, we also investigated the analgesic efficacy of inhaled ATPγS at different dosages (5, 10, and 15 mg kg^−1^). We found that 10 mg/kg produced a significant increase in mechanical and thermal thresholds (*p* = 0.0007 vs untreated SNI), while 15 mg kg^−1^ did not yield additional benefit (Figure S10A,B, Supporting Information). To assess systemic inflammation, we measured serum IL‐6 levels and observed no significant increase after inhalation of 10 mg kg^−1^ ATPγS (Figure S10C, Supporting Information). Moreover, we performed the inhalation of ATPγS in untreated mice and detected the thermal pain response in the hot plate test. ATPγS significantly increased thermal pain thresholds in these animals (*p* = 0.0137, Two‐tailed *t*‐test; Figure S10D, E). Consistently, c‐Fos expression in the vlPAG was also elevated following ATPγS administration (Figure S10F,G, Supporting Information). These findings reveal that the direct inhalation of P2rx2 agonist exhibits potent pain‐relieving properties under various conditions.

## 3 Discussion

In this study, we identified a neural pathway of the lung–brain axis to exert MSC‐induced analgesia. We found that stimulation of lung‐innervating *Npy2r* neurons improved neuropathic pain in the SNI mice, and these sensory neurons project to vlPAG, which might be the reason for vagal signaling‐related analgesia. Furthermore, MSC positively regulate *Npy2r* vagal sensory neurons via PANX1‐regulated extracellular ATP release to exert analgesic effects. Overall, this study reveals that specific sensory neurons respond to MSC infusion to alleviate pain and may lay the groundwork for aiming the body–brain interface in the treatment of neurological diseases.

MSC therapy has shown beneficial effects on diverse brain functions, including mood, cognition, and pain in rodent models.^[^ ^26^ ^]^ Building on previous work showing that intravenously delivered MSC regulate brain activity,^[^ ^15^ ^]^ we used whole‐brain clearing to map central activation and identified the vlPAG as a major responder. As a clinical target, the vlPAG has been used for deep‐brain stimulation (DBS) in refractory pain, but surgical risks limit its utility.^[^ ^27^ ^]^ Here, we provide evidence that MSC offer a noninvasive alternative by activating vlPAG through a lung vagal–brain circuit, leading to fast and effective analgesia. Comparative tests showed that MSC treatment produced analgesia similar to dexmedetomidine and superior to aspirin in the SNI model. Interestingly, we also observed sex‐specific differences in MSC analgesia, with male mice exhibiting greater improvements in mechanical pain thresholds than females. This may reflect known sexual dimorphisms in pain circuitry, particularly in the vlPAG, where dopamine neurons contribute to sex‐dependent modulation of nociception.^[^ ^28^ ^]^ We detected c‐Fos expression in the vlPAG region and found that MSC induced significantly higher c‐Fos signals in male mice than in female mice (*p* = 0.0022, one‐way ANOVA), as shown in Figure S11 (Supporting Information). These results point to sex‐dependent variation in vlPAG circuitry as a potential factor influencing MSC efficacy, deserving further study.

Next, our work found that peripheral MSC exert regulatory effects on distant brains mainly via vagal signaling. The vagus nerve serves as a critical sensory interface between the body and brain,^[^ ^29^ ^]^ and its activation has been associated with analgesia in various clinical and animal models.^[^ ^30^ ^]^ The lung, densely innervated by vagal fibers,^[^ ^31^ ^]^ represents a key target for this interface. While respiratory techniques have long been used to relieve pain,^[^ ^32^ ^]^ the underlying sensory pathways remained unclear. We demonstrated that *Npy2r* vagal sensory neurons projecting from the lung to the vlPAG contribute to pain modulation, offering mechanistic insight into lung–brain crosstalk. We further showed that MSCs specifically activate these *Npy2r* neurons, and that their activation is necessary for MSC‐induced analgesia. Given the cellular diversity of vagal ganglia,^[^ ^33^ ^]^ other sensory neuron subtypes may also mediate additional MSC effects. Mapping these body–brain pathways will be essential to fully understand the therapeutic potential of MSC in neurological disease.

Our findings also expand upon the well‐established literature showing that NPY and its receptors play key roles in endogenous pain inhibition, particularly at the spinal cord dorsal horn and brain. Y2R and Y1R in the spinal dorsal horn modulate pain by suppressing neurotransmitter release from primary afferents and dampening excitatory interneuron activity, respectively.^[^ ^34^ ^]^ Genetic or pharmacologic manipulation of these receptors alters pain sensitivity in both inflammatory and neuropathic models.^[^ ^35^ ^]^ Similarly, the NPY signaling and the function of NPY receptors in key pain‐related brain regions, including the parabrachial nucleus, periaqueductal gray, amygdala, and nucleus accumbens, also play an important role in the analgesia.^[^ ^36^ ^]^ NPY‐based therapeutic interventions targeting the central nervous system may represent a promising analgesic strategy. While we did not manipulate NPY pharmacologically, the identification of *Npy2r*‐expressing vagal sensory neurons projecting to the vlPAG suggests that our lung–brain circuit may represent a supraspinal extension of this conserved analgesic system.

In our previous work, we found that peripheral MSC modulate a lung vagal–brain pathway to alleviate depressive and anxiety‐like behaviors, highlighting the body–brain communication route. In both cases, MSC activated lung‐innervating vagal sensory neurons, which then projected to central brain regions via the NTS. However, the neurochemical identities of the vagal afferents and the target brain circuits differ between conditions. In the depression model, MSC activated 5‐HT neurons in the dorsal raphe nucleus, largely via BDNF‐TrkB signaling of sensory neurons in the nodose ganglia. Conversely, in the SNI model, analgesia was mediated through activation of *Npy2r*‐expressing pulmonary sensory neurons, which projected to the NTS and the ventrolateral periaqueductal gray (vlPAG)—a key hub in descending pain modulation. Compared to our previous work, this study provides a more clearly defined mechanistic pathway, including the identification of *Npy2r*‐expressing vagal sensory neurons and downstream vlPAG circuits, as well as a clinically translatable intervention via inhaled P2rx2 agonist, which altogether strengthens the therapeutic relevance of targeting the lung–brain axis in neuropathic pain.

Furthermore, we identified that MSC positively interact with *Npy2r* neurons via PANX1‐regulated extracellular ATP release. It has been published that ATP stimulates vagus nerves in the lung, resulting in pronounced bronchoconstriction mediated by P2rx2/3 receptors located on vagal sensory nerve terminals.^[^ ^37^ ^]^ Moreover, ATP is utilized as a neurotransmitter by lingual taste buds and airway epithelial cells for communication with vagal afferent nerves,^[^ ^22^, ^38^ ^]^ indicating the important role of ATP in signal transduction. Accordingly, our results showed that the inhalation of P2rx2 agonist, ATPγS exhibits analgesic effects in SNI mice. ATP has been shown to have a wide spectrum of unique pain‐relieving effects in various clinical situations, and such pain‐relieving effects are not very fast in onset,^[^ ^39^ ^]^ possibly because *Npy2r* sensory neurons are likely slow‐conducting C fibers.^[^ ^18^ ^]^ Although purinergic compounds may activate airway sensory pathways, our results and previous reports suggest that ATPγS is well tolerated within the tested dose range,^[^ ^40^ ^]^ supporting its potential as a non‐cell‐based therapeutic strategy pending further safety evaluation. Recently, numerous studies indicated that ATP may serve a vital cell–cell, cell–neuron, or neuron–neuron communication role in many organs. For instance, the sensation of extracellular ATP is an essential event defining bone marrow niches for plasma cells.^[^ ^41^ ^]^ Moreover, astrocyte Ca^2+^‐evoked ATP release regulates myelinated axon excitability and conduction speed.^[^ ^42^ ^]^ Considering the extensive beneficial and regulatory effects of MSC,^[^ ^43^ ^]^ ATP signaling also mediated the essential function of endogenous or injected exogenous MSC in future research.

Although our findings are primarily derived from rodent models, growing clinical and preclinical evidence supports the translational potential of MSC‐based therapies for pain management. A recent open‐label phase I/II study demonstrated that a single intravenous infusion of ExoFlo, a bone marrow‐derived MSC extracellular vesicle (EV) product, was safe and significantly improved pain scores and functional outcomes in patients with complex regional pain syndrome, a severe and treatment‐resistant chronic pain condition.^[^ ^11b^ ^]^ In parallel, preclinical studies have shown that MSC and their EV can reduce sensory neuron hyperexcitability and pain‐related behaviors in murine models of osteoarthritis, further supporting their analgesic potential through peripheral and central mechanisms.^[^ ^12^ ^]^ These findings highlight a promising avenue for developing MSC or MSC‐EV‐based therapeutics in chronic pain conditions. However, large‐scale, placebo‐controlled clinical trials are still required to optimize treatment protocols and confirm long‐term efficacy. Bridging the mechanistic insights from animal studies with clinical outcomes will be essential to facilitate the successful translation of MSC therapies to human pain management. As we used xenogeneic human MSC in immunocompetent mice, potential immune clearance may limit long‐term engraftment. Nonetheless, our previous study confirmed that human MSC retained their identity and viability within 72 h, which likely suffices for their short‐term paracrine and modulatory effects. Future studies will be needed to clarify longer‐term persistence under immune surveillance.

In conclusion, this study reveals the *Npy2r* sensory neuron‐mediated lung‐to‐brain pathway to activate vlPAG for MSC‐induced analgesic effects in the mouse model of neuropathic pain. Our findings may contribute to a better understanding of the body–brain connection and support the exploration of future pain‐management strategies.

## 4 Experimental Section

### Animals

All animal studies and experimental procedures were approved by the Sun Yat‐sen University Institutional Animal Care and Use Committee (SYSU‐IACUC‐2024‐B0004). For in vivo experiments, mice were maintained at the Experimental Animal Center of Sun Yat‐sen University, in accordance with the guidelines of Sun Yat‐sen University Institutional Animal Care and Use Committee. After all surgery, a moderate amount of lidocaine was added to the wound to relieve pain caused by the surgical procedures. Throughout all surgical procedures, the core temperature of all animals was maintained at 37 ± 0.5 °C with a heating pad. Animals recovered on a thermal pad from the procedure were returned to housing. Male and female C57BL/6J mice (6–14 weeks of age) were randomly assigned to experimental groups. All Mice were housed in four per cage under a 12‐h light–dark cycle (light on from 7 a.m. to 7 p.m., humidity between 30% and 70%, temperatures of 20 to 22 °C) with free access to food and water. All mouse lines are in a C57BL/6J background. Genotypes/sources for mice used in the above studies are listed in the Supporting Information.

### Spared Nerve Injury (SNI) Model

Surgeries were performed on wild‐type (C57BL/6J) or transgenic mice. Briefly, under isoflurane anesthesia, a 0.5‐cm skin incision was made on the left thigh to expose the three branches of the sciatic nerve (peroneal, tibial, and sural nerves). Common peroneal and tibial nerves were tightly ligated with a 6/0 silk suture and transected together. A 1‐mm piece of the nerves was removed. Precaution was taken to avoid damage to the sural nerve. After surgery, a drop of lidocaine was added to the wound to relieve pain caused by the surgical procedures. Then, the overlaying muscles and skin were separately closed with 6/0 silk and 4/0 Vicryl sutures, respectively. Throughout the surgical procedure, the core temperature of all animals was maintained at 37 ± 0.5 °C with a heating pad.

Animals recovered on a thermal pad from the procedure were returned to housing. In sham‐operated animals, exactly the same procedure was performed, but without ligation and transection of the nerves. Most of the SNI mice displayed neuropathic pain response (reduced thermal withdrawal latency and mechanical threshold) 1–2 weeks after SNI surgery. Animals that did not exhibit neuropathic phenotypes after 14 days were discarded without further experiments. Baseline measurements were taken before the surgeries.

### Chemotherapy‐Induced Neuropathic Pain

Paclitaxel (4 mg kg^−1^ day^−1^; MedChemExpress) was delivered by intraperitoneal injection (i.p.) every second day, adding up to a cumulative dose of 16 mg kg^−1^. The cytostatic paclitaxel was dissolved in a 1:9 (v/v) DMSO‐Corn Oil (Sigma) solution and stored at −80 °C until further usage.

### Complete Freund's Adjuvant (CFA)‐Induced Pain

CFA (Sigma) was injected (10 µL) into the plantar surface of the hindpaw after a baseline VFT or HPT.

### Cell Sources, Culture, and Administration

Bone marrow‐derived mesenchymal stromal cells (MSC) and human dermal fibroblasts (HDF) used in this study have been approved by the Institutional Review Board of Sun Yat‐Sen University. Human cells were isolated and/or cultured as follows:

### Cell Sources, Culture, and Administration—MSC

Heparin‐treated bone marrow was obtained by iliac crest aspiration from healthy donors following the Declaration of Helsinki protocols with informed consent, and the protocol was approved by the relevant Ethics Review Board prior to initiation. In brief, bone marrow mononuclear cells were obtained by Ficoll‐Hypaque (1.077 g mL^−1^, Amersham Biosciences) density gradient centrifugation and seeded into 75‐cm^2^ flasks (Corning) in medium consisting of low‐glucose DMEM (L‐DMEM, HyClone) and 10% fetal bovine serum (FBS, HyClone). After 3 days of culture, the medium was replaced, and non‐adherent cells were discarded. At 70‐80% confluence, these cells were harvested by trypsin and cultured at 1 × 10^4^ cells cm^−2^ in 75‐cm^2^ flasks and designated as passage 1. These cells were further passaged at a ratio of 1:3. According to our previous studies, these cells expressed the surface markers CD29, CD44, CD73, CD90, CD105, and CD166, but not the hematopoietic markers CD45 and CD34. At the 5th passage, the multipotent differentiation capacity of MSC was confirmed by the induction of adipogenic, osteogenic, and chondrogenic phenotypes. These well‐characterized sixth passage MSC were used in both in vitro and in vivo studies. GFP‐MSC and RFP‐MSC were transduced by separately introducing enhanced green fluorescent protein (EGFP) and red fluorescent RFP lentiviral expression vectors, according to our previous studies.^[^ ^15^, ^44^ ^]^

### Cell Sources, Culture, and Administration—HDF

A 1 cm^2^ piece of skin was placed on culture dishes and incubated in an MF‐start medium (Toyobo) for 5 days. Cells that migrated out of the graft pieces were transferred to new plates and maintained in DMEM containing 10% FBS.

Cells (1×10^6^ per mouse) were resuspended in 200 µL of 0.9% saline and delivered via tail‐vein injection. Before each injection, the saline solution containing cells was thoroughly shaken to ensure that the cell fluid does not clump and avoid potential blockage risks.

### MSC‐Derived Extracellular Vesicles (EV) Isolation and Injection

MSC for EV isolation were cultured in serum‐free culture medium (GIBCO StemPro MSC SFM XenoFree medium) for three days. The conditioned medium was then collected and centrifuged at 300 g for 5 min, with supernatant transferred to a falcon tube for further centrifugation at 2000 g for 20 min at 4 °C. Supernatant was then transferred into polycarbonate ultracentrifuge tubes (Beckman, USA) for differential sequential ultracentrifugation at 10 000 g for 45 min and 100 000 g for 90 min. The collected pellet was resuspended in PBS for further ultracentrifugation at 100 000 g for 90 min. Newly collected pellet was resuspended in PBS and stored at −80 °C for use. Each mouse was injected with EV extracted from the supernatant of MSC (1 × 10^6^) cultured for three days.

### Assessment of Mechanical Allodynia

Mechanical allodynia was assessed with the up–down method. In brief, after habituation, a series of von Frey hairs with varying forces was applied to the lateral plantar surface of the hind paw of mice. Filaments were applied in either ascending or descending strengths to determine the filament strength closest to the hind paw withdrawal threshold. Each filament was applied for a maximum of 2 s at each trial. Quick withdrawal or licking of the paw in response to the stimulus was considered a positive response. Tactile threshold for each hind paw was then calculated according to the commonly used method.

### Hot Plate Test (HPT)

Mice were habituated to plexiglass chambers on top of a hot plate for 5 min. The hot plate was heated to 55 °C. The mice were placed on the hot plate, and the latency to withdraw was recorded by skilled researchers blinded to the experimental conditions.

### Elevated Plus Maze (EPM)

The elevated plus maze consisted of two opposite open arms (28.5 cm × 7 cm), two opposite closed arms (28.5 cm × 7 cm), and one central zone (7 cm × 7 cm). The plus‐shaped apparatus was elevated 55 cm above the ground. During the test, mice were placed in the open arm of the maze, facing the center zone, and a 10‐min monitoring of behavior was performed using a SONY HDR‐CX405 video camera. The time and entry numbers in the open arms were quantified and analysed by behavioral analysis software purchased from Clever Systems.

All behavioral tests were performed during the light cycle in a dedicated soundproof behavioral facility; assessments were performed by experimenters blind to treatment. Mice were brought to the testing room 30 min before the start of each behavioral test and remained in the same room throughout the test. After each behavioral test, the instruments were cleaned with 75% ethanol.

### Tail Suspension Test (TST)

Mice were suspended by the tail, which was taped and secured to a horizontal bar 30 cm above the floor, which ensured that the mice could not make any other contact or climb during the assay. Animal behaviors were videotaped for 6 min using a SONY HDR‐CX405 video camera. The animals were habituated for the first 1 min, and the time spent immobile during the subsequent 5‐min test period was counted offline by an observer blinded to animal treatment.

### CUBIC Whole‐Brain Clearing and Imaging

The clearing of the fixed tissue samples was conducted with Nuohai Tissue Clearing Kit (Nuohai Life Science (Shanghai) Co., Ltd), according to the manufacturer's instructions.

3D fluorescence imaging of the cleared tissues was conducted with a Nuohai LS 18 Tiling Light Sheet Microscope (Nuohai Life Science (Shanghai) Co., Ltd). A 4‐tile tiling light sheet was used to illuminate the sample, and a 1×/0.25 NA objective (Olympus MVPLAPO) was used to collect the fluorescence. The magnification of the microscope was set at 4×, and the spatial resolution was roughly 3.3 × 3.3 × 7 µm^3^ at the selected imaging conditions. The collected images were processed with the LS 18 ImageCombine software (Nuohai Life Science (Shanghai) Co., Ltd) and rendered using Amira (Thermo Fisher Scientific, USA).

### Stereotaxic Surgery

For intracranial injection, mice were anesthetized with isoflurane (3% during induction, 1.5% during maintenance, flow rate = 2 L min^−1^) and fixed on a stereotaxic device (RWD Life Science) equipped with an electronic heating pad. The scalp was cut, and a 10‐mm incision was made posterior to the bregma along the midline. Injections were performed with a Hamilton 1.0‐µL syringe needle (Hamilton 7000 series) and a microsyringe pump (KDS Legato 100, Sigma–Aldrich) at a rate of 100 nL min^−1^. After injection, the glass pipette was left at the site for 5 min and then slowly withdrawn. The scalp was sutured, and the mouse was allowed to fully recover from anesthesia and returned to its home cage. Throughout the surgical procedure, the core temperature of all animals was maintained at 37 ± 0.5 °C with a heating pad. All viral vectors and drugs were subdivided into aliquots and stored at −80 °C until use, and were listed in the key resources table.

### Virus Injection

To specifically eliminate the vlPAG neurons, the rAAV‐hSyn‐taCasp3 purchased from BrainVTA (Wuhan, China) was injected into the vlPAG (Anterior‐posterior (AP): ‐4.6‐4.7 mm; Medial‐lateral (ML): ±0.5 mm; Dorsal‐ventral (DV): ‐3.3mm). The injection volume of the AAV per mouse was 500 nL.

For retrograde tracing of vagal nerves innervating the lung, the rAAV2‐retro‐DIO‐EGFP purchased from BrainCase (Wuhan, China) was delivered to *VGLUT2‐Cre* mice by intratracheal injection. The injection volume of the AAV per mouse was 40 µL.

For anterograde tracing of NTS neurons, the HSV‐EGFP purchased from BrainVTA (Wuhan, China) was injected into the NTS (Anterior‐posterior (AP): ‐7.5 mm; Medial‐lateral (ML): ±0.3 mm; Dorsal‐ventral (DV): ‐4.5 mm). The injection volume per mouse was 500 nL.

To specifically induce the activation of *Npy2r* sensory neurons innervating the lung, the rAAV2‐retro‐DIO‐hM3D(Gq)‐mCherry purchased from BrainVTA (Wuhan, China) was delivered by intratracheal injection to *Npy2r‐Cre* mice. The injection volume of the AAV per mouse was 40 µL. After virus expression, the DREADD agonist clozapine N‐oxide (CNO) was injected intraperitoneally (1 mg kg^−1^). Moreover, the rAAV2‐retro‐DIO‐ChRmine‐EYFP purchased from BrainVTA (Wuhan, China) was delivered by intratracheal injection to *Npy2r‐Cre* mice. The injection volume of the AAV per mouse was 40 µL. After virus expression, a custom‐made 591 nm LED light was used to stimulate the mice's lungs through the skin.

To specifically induce the activation of P2ry1 vagal nerves innervating the lung, the mixture (1:1) of rAAV2‐retro‐DIO‐hM3D(Gq)‐mCherry and rAAV2‐retro‐P2ry1‐Cre customized from BrainVTA (Wuhan, China) was delivered by intratracheal injection to mice. The injection volume of the AAV per mouse was 40 µL. After virus expression, the DREADD agonist clozapine N‐oxide (CNO) was injected intraperitoneally (1 mg kg^−1^).

For tracing *Npy2r* vagal nerves innervating lungs‐NTS‐vlPAG pathways, the rAAV2‐retro‐DIO‐mCherry purchased from BrainVTA (Wuhan, China) was delivered by intratracheal injection to *Npy2r‐Cre* mice (Injection volume: 40 µL per mouse). After three weeks, PRV‐EGFP purchased from BrainVTA (Wuhan, China) was injected into the vlPAG (Injection volume: 500 nL per mouse).

To specifically inhibit *Npy2r* sensory neurons innervating the lung, the rAAV2‐retro‐DIO‐hM4D(Gi)‐EGFP purchased from BrainVTA (Wuhan, China) was delivered by intratracheal injection to *Npy2r‐Cre* mice. The injection volume of the AAV per mouse was 40 µL. After virus expression, the CNO was injected intraperitoneally (1 mg kg^−1^).

### Vagotomy

Mice were anesthetized with isoflurane, and a longitudinal midline incision was made in the ventral region of the neck before blunt dissection. The overlying muscles and fascia were separated to reveal the left or right vagal nerves. For the vagotomy group, the vagus was carefully stripped away from the carotid artery and precisely cut. For the sham group, the vagus was kept intact. After surgery, a drop of lidocaine was added to the wound to relieve pain caused by the surgical procedures. Then, the overlaying muscles and skin were separately closed with 6/0 silk and 4/0 Vicryl sutures, respectively. Throughout the surgical procedure, the core temperature of all animals was maintained at 37 ± 0.5 °C with a heating pad.

### Fast Blue Tracing

Mice were positioned on the surgical platform and anesthetized with isoflurane. Fast blue was delivered by intratracheal injection at a concentration of 5% weight/volume in water. The injection volume per mouse was 3 µL.

### In Vivo Electrophysiology (vlPAG) and Data Analysis

For in vivo electrophysiology recording, a custom‐made electrode (Yige Biotechnology Co., Ltd., Jiangsu, China) was inserted slowly toward the vlPAG (AP: ‐4.6 mm; ML: 0.5 mm; DV: ‐3.3 mm) of mice. Three screws were embedded in the skull, and stainless steel wires were wound around the skull‐penetrating screws as a ground. After surgery, mice were individually housed for at least 1 week. Local field potential (LFP; digitized at 1 kHz, low‐pass filtered up to 250 Hz) and spontaneous spiking activity (digitized at 40 kHz, band‐pass filtered between 300 and 6000 Hz) were recorded. Data were recorded for 30 min before (baseline) and after 591 nm LED light on the chest of mice. All data recorded from each microelectrode were imported into Offline Sorter V4 (Plexon Inc.). Single units were manually identified by threshold‐crossing and principal component analysis (PCA). Data analysis was conducted using Neuroexplorer 5 (Plexon Inc.) and MATLAB.

### Fiber Photometry Recording

Mice were injected with rAAV‐hSyn‐GCaMP6 in vlPAG, and the vlPAG was implanted with an optical fiber (0.2 mm O.D., 0.37 mm numerical aperture (NA); Inper Ltd., China) with a ceramic ferrule (AP: ‐4.6 mm; ML: 0.5 mm; DV: ‐3.3 mm). The ceramic ferrule was supported with a screw and dental cement. Mice were housed for 3 weeks to allow for virus expression and recovery, and then a fiber photometry system (Nanjing Thinkertech) was utilized to record calcium activity in the vlPAG upon ChRmine‐mediated stimulation of *Npy2r* sensory neuron innervating lungs. Photometry data were exported as MATLAB files for further analysis. Changes in fluorescence intensity were expressed as percentage changes over baseline (ΔF/F). F means were determined from the average fluorescence intensity during recording.

### Immunofluorescence Analysis

Mice were transcardially perfused with PBS followed by 4% paraformaldehyde. Following perfusion, tissues were harvested and kept in 4% paraformaldehyde for 6–24 h and then in 30% sucrose solution for 24–48 h. The tissues were then frozen and cut into 40‐µm sections. The tissue sections were incubated with 5% normal goat serum (Sigma) and 0.1% Triton X‐100 (HyClone) in PBS for 1 h at room temperature, followed by incubation with primary antibodies at 4 °C overnight. Next, the sections were incubated with secondary antibodies at room temperature for 1 h. All used antibodies are listed in the Supplementary Information.

### c‐Fos Measurement

To determine the effects of MSC on brain neuronal activity, mice were sacrificed 3 h after MSC injection and perfused as described above. Brain samples were collected for CUBIC whole‐brain clearing and imaging. Moreover, brain frozen sections were also obtained, and the expression of c‐Fos was detected by immunofluorescence to visualize neuronal activation in the vlPAG and NTS. To detect the activation of neurons in the nodose ganglia, mice were sacrificed after MSC injection (1 h/2 h/3 h) and perfused as described above, and the vagus nerve was separated from the carotid artery until the nodose ganglia became accessible. To visualize neuronal activation, the expression of c‐Fos was detected by immunofluorescence of the whole nodose ganglia.

### Flow Cytometry

As for the isolation of infused GFP‐MSC from mouse lungs, GFP‐MSC (1 × 10^6^ per mouse) were resuspended in 200 µL of 0.9% saline and delivered via tail–vein injection to mice. After 3 days, the collected lungs were minced into small pieces with 5 mL of HBSS digestive solution, including Type IV collagenase, and incubated in a water bath at 37 °C for 30 min. After adding DMEM containing 10% fetal bovine serum (FBS, HyClone) to stop digestion, the cell suspensions were centrifuged at 1250 × g for 5 min at room temperature, washed twice with phosphate‐buffered saline (PBS), and filtered through a 40‐µm cell strainer to prepare a single‐cell suspension. Then, a flow cytometry assay was used to isolate GFP‐MSC from the single‐cell suspension. CytoFLEX flow cytometer was used to perform flow cytometry experiments, and the results were assessed with the CytExpert (Beckman) and FlowJo X 10.0.7r2 software packages (BD). The LIVE/DEAD(Invitrogen) was utilized for the cell viability detection according to the manufacturer's manual.

### Calcium Imaging and Analysis—Lung imaging ex vivo

Mice (*Npy2r‐Cre; Loxp‐GCaMP6*) were anesthetized with isoflurane, and MSC (1 × 10^6^ per mouse) or PBS were delivered via tail–vein injection. After 30 min, fresh lungs were dissected from mice, and placed in a perfusion imaging chamber (Warner Instruments) filled with physiological buffer (125 mm NaCl, 5.9 mm KCl, 2.56 mm CaCl_2_, 1 mm MgCl_2_, 25 mm HEPES, 0.1% BSA, pH 7.4). Then, the lungs were imaged on the FVMPE‐RS multiphoton microscope (Olympus).

### Calcium Imaging and Analysis—Nodose Imaging In Vivo

Mice were anesthetized with isoflurane, and MSC (1 × 10^6^ per mouse) or PBS were delivered via tail–vein injection. After 30 min, the right vagal ganglion was exposed by blunt dissection and imaged on the FVMPE‐RS multiphoton microscope (Olympus). Specifically, the vagus nerve was carefully separated, and the cervical artery was ligated with black silk thread to avoid bleeding. Follow the vagus nerve upward until the ganglion is exposed, and the water‐soluble imaging gel (Tianjin Xiyuan Temple Production Institute) was carefully added to the nodose ganglion. Then, the mouse was placed on the stage, and the nodose ganglion was moved to the center of the field of view under a 5× lens. Next, a 20× water lens was immersed in the gel for imaging.

### Calcium Imaging and Analysis—Lung‐Innervating Vagal Sensory Neuron Imaging

To specifically image *Npy2r* sensory neurons innervating the lung, rAAV‐retro‐DIO‐GCaMPs purchased from BrainVTA (Wuhan, China) was delivered by intratracheal injection to *Npy2r‐Cre* mice. The injection volume of the AAV per mouse was 40 µL. After virus expression, the nodose imaging was performed as shown above.

### Calcium Imaging and Analysis—Nodose Imaging In Vitro

Fresh nodose ganglia were collected from *Npy2r‐Cre; Loxp‐GCaMP6* mice and cultured in the Neurobasal medium (Gibco) with or without RFP‐MSC for several hours. The cultured nodose ganglia were imaged for several hours on the live cell imaging analysis system (BioTek lionhert FX).

The GCaMP6 fluorescence intensity of each frame was quantified by Image J software (https://imagej.nih.gov/ij/). Changes in fluorescence intensity are expressed as the percentages of change over baseline (ΔF/F).

### Drug Treatment

Mice were treated with aspirin orally (200 mg kg^−1^). Dexmedetomidine was delivered by intraperitoneal injection (i.p. 30 µg kg^−1^). P2rx2/P2rx3 antagonist, minodronic acid (YM‐529, 10 mg kg^−1^) was delivered via inhalation treatment before MSC injection. ATPγS (10 mg kg^−1^) was delivered via inhalation treatment to SNI mice.

### Biochemical Examination

Mice were anesthetized with isoflurane at the indicated time points, and blood was collected via cardiac puncture and centrifuged at 600 g for 15 min to obtain serum. Fresh mouse lungs were removed, homogenized in PBS containing protease and phosphatase inhibitors (Roche), and centrifuged at 10 000 g for 10 min. The supernatant and serum were collected and immediately stored at −80 °C until use. Commercial ELISA kits were used for quantitative detection of IL‐6 (Invitrogen, 88‐7064) in the serum. A commercial ATP detection assay kit was used for the quantitative detection of ATP (Servicebio, G4309) in the lung tissue homogenates and cell supernatants.

### **RNA** Interference **(RNAi)**

To knock down Pannexin1 (PANX1) in MSC, the PANX1 interference vectors were synthesized from RIBOBIO (Guangzhou, China). MSCs were seeded into six‐well plates and transfected with interference vectors using Lipofectamine RNAiMAX reagent (Invitrogen). The RNAi sequences are presented in the Supplementary Information.

### RNA Isolation and Quantitative Real‐Time and Reverse Transcription PCR

Total RNA was extracted using the TRIzol reagent (Invitrogen), and 1 µg of RNA was reverse transcribed using a RevertAid First Strand cDNA Synthesis Kit (Thermo Scientific). The generated cDNA was subjected to real‐time PCR with the SYBR Green reagent (Roche). The primer sequences are listed in the Supplementary Information.

### Western Blot Assay

MSC lysates were prepared with RIPA lysis buffer (Sigma) containing phosphatase and protease inhibitor cocktails (Roche) and centrifuged at 4 °C. Supernatants were collected and immediately stored at −80 °C until use. Proteins were separated by SDS‐PAGE, transferred to a polyvinylidene fluoride (PVDF) membrane, blocked with 5% non‐fat milk, incubated overnight with the appropriate primary antibody at 4 °C, and then incubated with secondary antibodies at room temperature. All of the utilized antibodies are listed in the Supplementary Information.

### RNA Sequencing

Total RNA was extracted using a Trizol reagent kit (Invitrogen, Carlsbad, CA, USA) according to the manufacturer's protocol. RNA quality was assessed on an Agilent 2100 Bioanalyzer (Agilent Technologies, Palo Alto, CA, USA) and checked using RNase‐free agarose gel electrophoresis. After total RNA was extracted, eukaryotic mRNA was enriched by Oligo(dT) beads. Then the enriched mRNA was fragmented into short fragments using the fragmentation buffer and reverse transcribed into cDNA by using NEBNext Ultra RNA Library Prep Kit for Illumina (NEB# 7530, New England Biolabs, Ipswich, MA, USA). The purified double‐stranded cDNA fragments were end‐repaired, A base added, and ligated to Illumina sequencing adapters. The ligation reaction was purified with the AMPure XP Beads (1.0X). Polymerase chain reaction (PCR) amplified. The resulting cDNA library was sequenced using Illumina Novaseq6000 by Gene Denovo Biotechnology Co. (Guangzhou, China). The mapped reads of each sample were assembled by using StringTiev1.3.1 in a reference‐based approach. For each transcription region, a FPKM (fragment per kilobase of transcript per million mapped reads) value was calculated to quantify its expression abundance and variations, using RSEM software.

### Statistical Analysis

All summarized data are expressed as the mean ± SD. In the box plot, lower and higher bounds, central line, lower and higher whiskers, respectively, represent 25th, 75th, 50th, 5th, and 95th percentiles. Statistical comparisons were made with Prism software (v 8.02, GraphPad) using the two‐tailed Student's *t*‐test (between two groups) or the one‐way ANOVA (for multigroup comparisons) as appropriate. Before performing one‐way ANOVA or *t*‐test, the normality and homogeneity of variances were tested. When the normality assumption was violated, the Kruskal‐Wallis test (the nonparametric equivalent of the one‐way ANOVA) or the Mann‐Whitney U test was performed. Moreover, when the homogeneity of variances was violated, the Brown‐Forsythe ANOVA test was performed.

Mice were randomized into control or treatment groups. Detailed information on statistical tests and sample sizes is indicated in the figure legends. Specifically, *n* = 3 mice or 3 biologically independent samples: Figure 5C,I, Figure 6A,H; *n* = 4 mice or biologically independent samples: Figure 3N, Figure 6F,G,O; *n* = 5 mice or biologically independent samples: Figure 1B, Figure 2I,K,N, Figure 3F–J, Figure 4C,D, Figure 6E,N, Figure 7B–D; *n* = 6 mice or biologically independent samples: Figure 1P; *n* = 8 mice or biologically independent samples: Figure 1C–F,I,L,Q, Figure 2N, Figure 3L, Figure 4F,G; *n* = 9 mice or biologically independent samples: Figure 7F; *n* = 10 mice or biologically independent samples: Figure 1H, 7E. All experiments involved biological (not technical) replicates. All representative micrographs are from three independent experiments. ^*^ *p* < 0.05, ^**^ *p* < 0.01, ^***^ *p* < 0.001, ^****^ *p* < 0.0001, ns‐no significant difference.

## Conflict of Interest

The authors declare no conflict of interest.

### Author Contributions

J.H., T.Z., and Y.S. contributed equally to this work. J.H. conceived, performed, and analyzed the majority experiments in this study. T.H.Z., Y.W.D., and K.Y.C. performed behavioral tests and the assessment of mechanical allodynia. Y.M.S., Y.C.M., and R.J.L. generated and analysed RNA sequencing data. Y.Q., T.W., and X.Y.C. performed the surgery of SNI. L.H. isolated the MSC‐EV. L.Z. performed the cell culture of MSC. X.Y.T. performed the identification of genetic mice. Y.W., Z.M.L., and R.C.L. performed brain section staining and microscopy with the help of Y.L.T. and Y.T.L. H.S.L. and N.L. performed the inflammatory cytokine detection. R.F., J.Q.F., and X.H.W. contributed confocal images of MSC distribution and discussed results. A.P.X. and X.R.Z. conceived and designed the study. A.P.X., X.R.Z., and J.H. wrote the original manuscript. X.F. revised the manuscript and provided a lot of experimental guidance in the revision. A.P.X. and X.R.Z. supervised the whole project.

## Supporting information



Supporting Information

## Data Availability

All data supporting the findings of this study are available from the corresponding author upon request. Source data are provided with this paper. No custom algorithms were used in this study. The raw sequence data reported in this paper have been deposited in the Gene Expression Omnibus with ID (GEO: GSE246290, GSE305716) that are publicly accessible at https://www.ncbi.nlm.nih.gov/gds.
