# Supplementary material for: Mesenchymal Stromal Cells Play an Analgesic Role Through a Npy2r Sensory Neuron‐Mediated Lung‐to‐Brain Axis
Source: Adv Sci (Weinh). 2025 Aug 28;12(43):e04922. doi: 10.1002/advs.202504922 (PMC12631818; doi:10.1002/advs.202504922)
Supplement: Supplementary file 1 — Supporting Information [file ADVS-12-e04922-s001.docx]

**Supplementary Information**

**Contents**

**Supplementary Fig. 1** MSC exhibit analgesic effects in the mouse pain models. (related to Figure 1).

**Supplementary Fig. 2** MSC infusion regulates vlPAG (related to Figure 1).

**Supplementary Fig. 3** MSC positively regulate NTS via vagal signaling (related to Figure 2).

**Supplementary Fig. 4** The distribution of lung-innervating *Npy2r* and *P2ry1* sensory neurons (related to Figure 3).

**Supplementary Fig. 5** The activation of lung-innervating *Npy2r* sensory neurons increases vlPAG neuron activity and reduces pain. (related to Figure 3).

**Supplementary Fig. 6** The neural tracing of lung-to-brain pathway (related to Figure 3).

**Supplementary Fig. 7** The colocalization between *Npy2r* and TUBB3 expression in lungs (related to Figure 4).

**Supplementary Fig. 8** MSC activate the lung-innervating *Npy2r* sensory neurons (related to Figure 5).

**Supplementary Fig. 9** MSC exert regulatory effects on lung-to-brain axis for analgesia via PANX1-mediated ATP signaling (related to Figure 6).

**Supplementary Fig. 10** The inhalation of ATPγS improves pain threshold (related to Figure 7).

**Supplementary Fig. 11** MSC induce the activation of vlPAG neurons in male and female mice (related to Discussion).

**Supplementary Table 1** Antibodies.

**Supplementary Table 2** Viruses

**Supplementary Table 3** Regents and kits

**Supplementary Table 4** Mouse strains

**Supplementary Table 5** Oligonucleotides

**Supplementary sequences information**


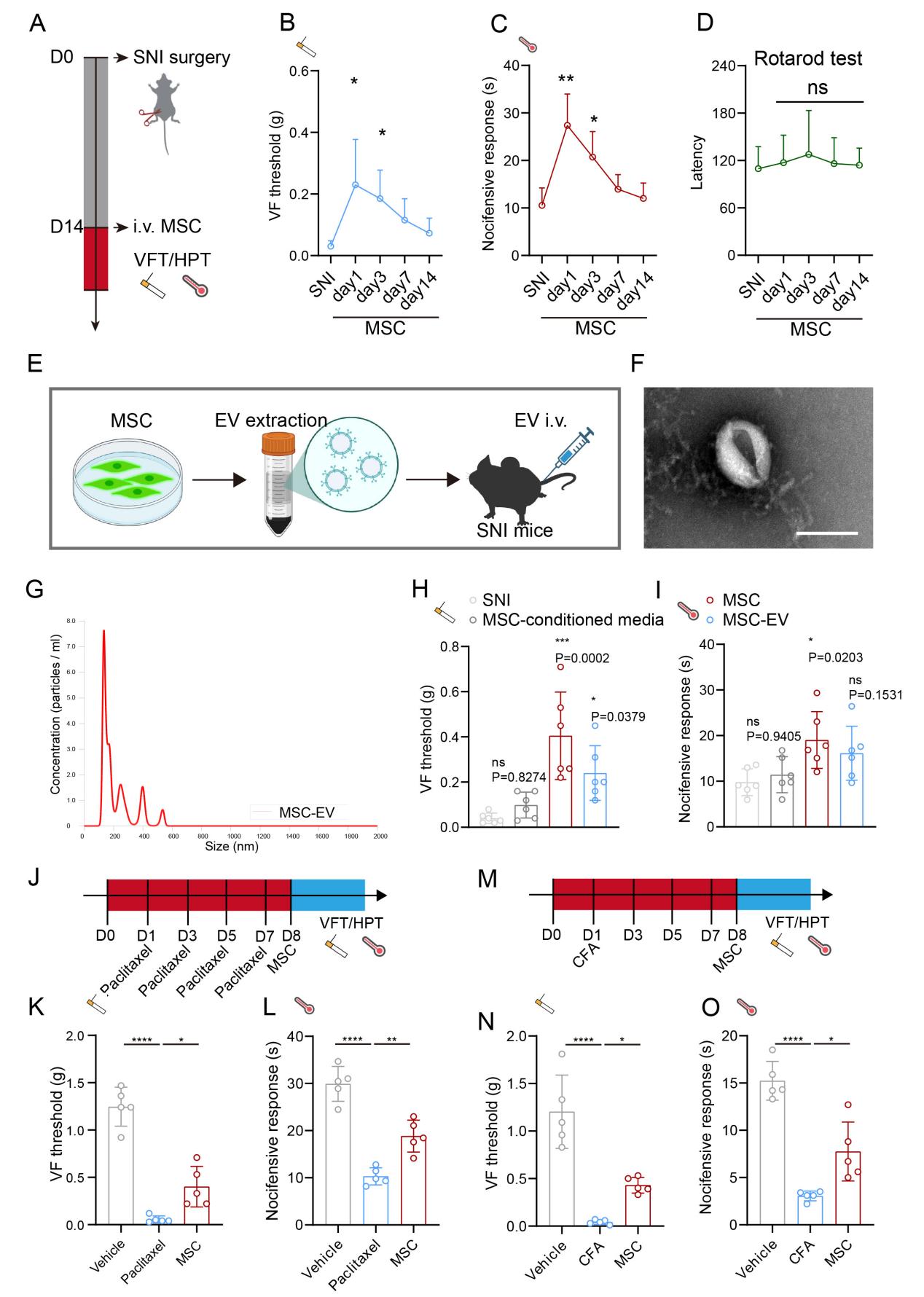


**Supplementary Figure 1. MSC exhibit analgesic effects in the mouse pain models. Related to Figure 1.**

1. Schematic diagram showing the experimental procedures used for MSC therapy in SNI mice.
2. The change of paw withdrawal threshold assessed by the VFT 1 day, 3 days, 7 days, and 14 days after MSC infusion. Two-tailed t test. n = 4 mice.
3. The change of paw withdrawal latency assessed by the HPT 1 day, 3 days, 7 days, and 14 days after MSC infusion. Two-tailed t test. n = 4 mice.
4. Rotarod motor testing results. Two-tailed t test. n = 4 mice.
5. Schematic diagram showing the experimental procedures used for MSC-derived extracellular vesicle (EV) therapy in SNI mice.
6. Representative image showing the MSC-EV in vitro. Scale bar, 100 nm
7. Representative nanoparticle tracking analysis (NTA) analysis of EV isolated from MSC.
8. Paw withdrawal threshold assessed by the VFT after 4 hours of MSC-conditioned media, MSC, and MSC-EV injection in SNI mice. n = 6 mice. One-way ANOVA.
9. Paw withdrawal latency assessed by the HPT after 4 hours of MSC-conditioned media, MSC, and MSC-EV injection in SNI mice. n = 6 mice. One-way ANOVA.
10. Schematic diagram showing the experimental procedures used for MSC therapy in chemotherapy induced peripheral neuropathy pain model by Paclitaxel.
11. Paw withdrawal threshold assessed by the VFT after 4 hours of MSC injection in the chemotherapy-induced pain model. n = 5 mice. One-way ANOVA.
12. Paw withdrawal latency assessed by the HPT after 4 hours of MSC injection in the chemotherapy-induced pain model. n = 5 mice. One-way ANOVA.
13. Schematic diagram showing the experimental procedures used for MSC therapy in complete Freund's adjuvant (CFA)-induced inflammatory pain model.
14. Paw withdrawal threshold assessed by the VFT after 4 hours of MSC injection in the CFA-induced pain model. n = 5 mice. One-way ANOVA.
15. Paw withdrawal latency assessed by the HPT after 4 hours of MSC injection in the CFA-induced pain model. n = 5 mice. One-way ANOVA.

Illustrations created with BioRender.com. ^*^P < 0.05, ^**^P < 0.01, ^***^P < 0.001, ^****^P < 0.0001, ns - no significant difference. Error bars indicate the SD.

**
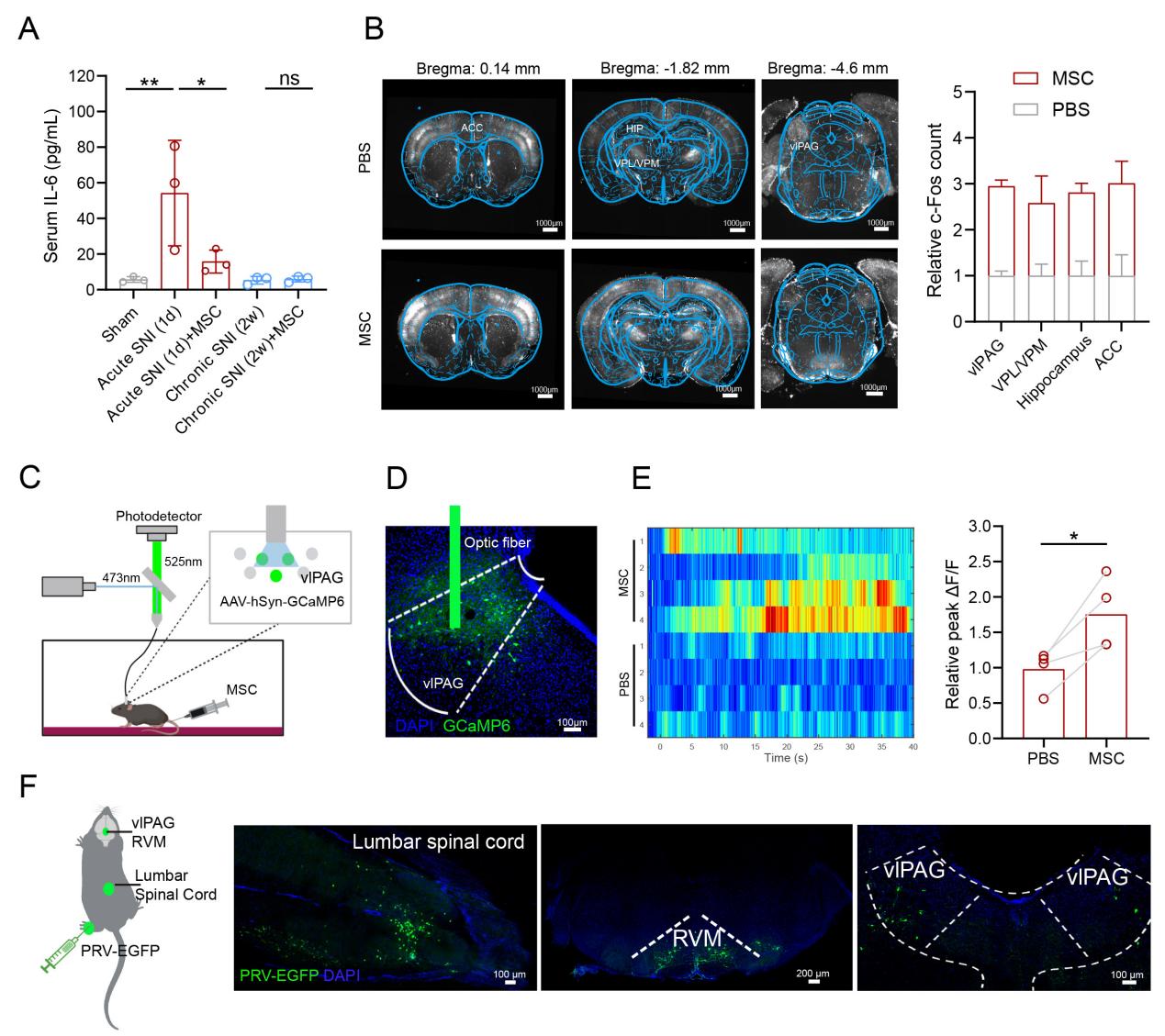
**

**Supplementary Figure 2. MSC infusion regulates vlPAG. Related to Figure 1.**

1. Enzyme-linked immunosorbent assay - quantified serum concentration of interleukin (IL)-6 (n = 3 mice). One-way ANOVA.
2. CUBIC whole-brain Fos imaging results. Scale bar, 1000 μm. Ventral lateral parts of periaqueductal gray area (vlPAG), hippocampus (HIP), ventral posterolateral (VPL) and ventral posteromedial (VPM) thalamus, anterior cingulate cortex (ACC).
3. Schematic diagram for optic fiber recordings in vlPAG after MSC injection.
4. Representative image showing GCaMP6 expression in vlPAG.
5. Left: Heatmaps of spontaneous calcium signals. Right: Statistical analysis showed relative peak fluorescence intensity changes (ΔF/F) in vlPAG (n = 4 mice). Two-tailed t-test.
6. Transsynaptic labeling of PRV-EGFP. Injection site: hindpaw. Rostral ventromedial medulla (RVM).

Illustrations created with BioRender.com. ^*^P < 0.05, ^**^P < 0.01, ^***^P < 0.001, ^****^P < 0.0001, ns - no significant difference. Error bars indicate the SD.

**
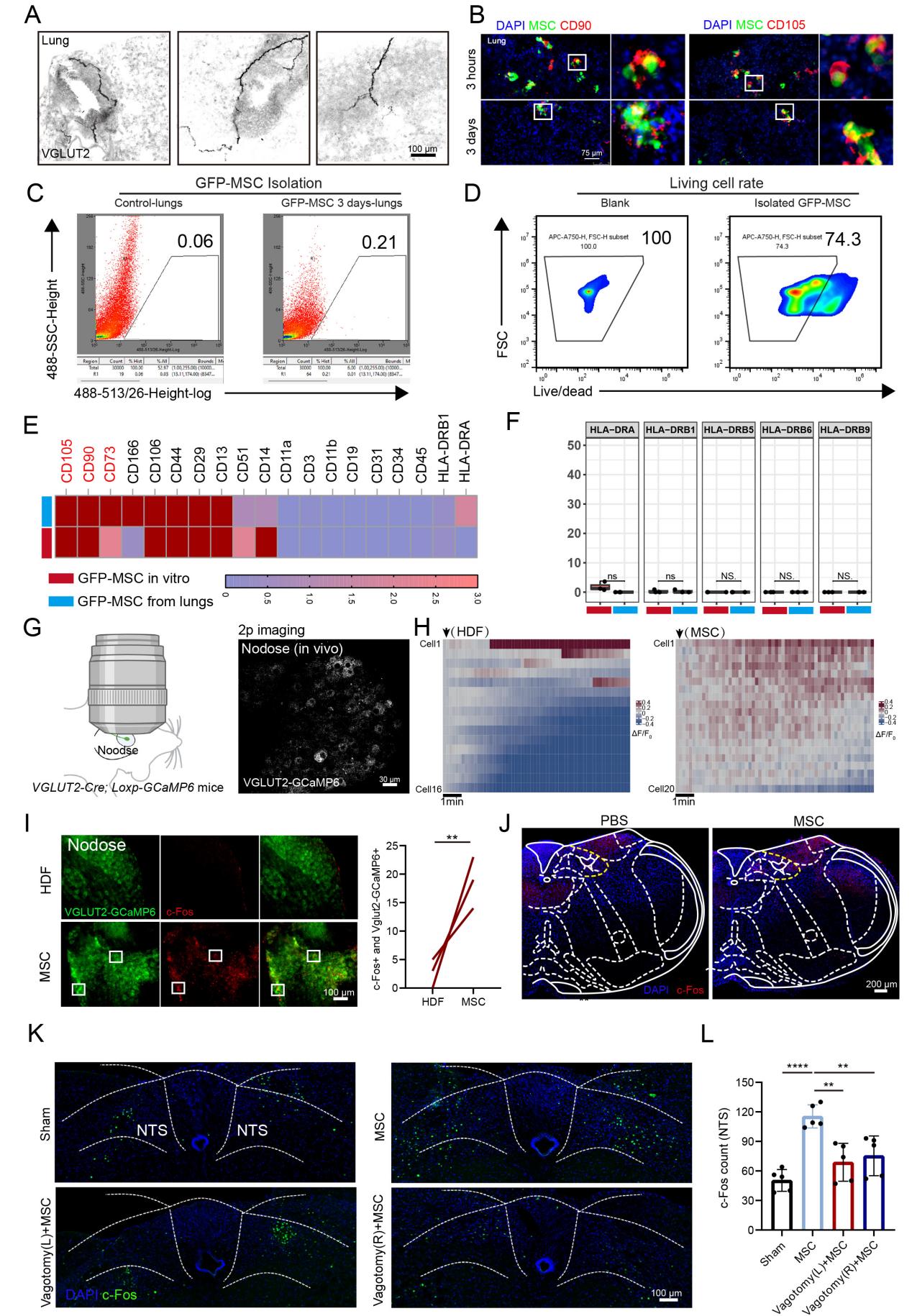
**

**Supplementary Figure 3. MSC positively regulate NTS via vagal signaling. Related to Figure 2.**

1. Representative images of terminals formed by VGLUT2 sensory neurons innervating lungs. Scale bar, 100 μm.
2. Representative immunofluorescence images of the expression of MSC specific markers of infused GFP-MSC in lungs. Scale bar, 75 µm.
3. The flow cytometry sorting of GFP+ cells from lung homogenates in mice after three days of GFP-MSC infusion.
4. The flow cytometric analysis was performed to detect the cell viability of isolated GFP+ cells from mice lungs by using LIVE/DEAD™ (Invitrogen).
5. Heat map showing that GFP-MSC in vitro or GFP-MSC isolated from lungs had similar expression profiles for MSC-related surface marker-encoding genes.
6. Box plot showing that GFP-MSC in vitro and GFP-MSC isolated from lungs barely expressed HLA-DR.
7. Left: cartoon depicting in vivo vagal ganglion imaging after MSC or HDF injection in the *VGLUT2-Cre; Loxp-GCaMP6* mice. Right: representative images of GCaMP6 fluorescence in vagal ganglion. 2p imaging, two photon imaging. Scale bar, 30 μm.
8. Heatmap depicting vagal sensory neuron calcium responses to HDF (ΔF/F, 16 imaged neurons) or MSC (ΔF/F, 20 imaged neurons).
9. Left, representative images showing the c-Fos expression in nodose ganglia of *VGLUT2-Cre; Loxp-GCaMP6* mice. Scale bar, 100 μm. Right: statistical analysis of VGLUT2-GCaMP6 and c-Fos colocalization in nodose ganglia after MSC or HDF injection (n = 3 mice). Two-tailed t-test.
10. Representative images showing the c-Fos expression in the brainstem (Bregma: -7.56 mm). Scale bar, 200 μm.

(K and L) Representative images (K) and statistical analysis (L) showing the NTS c-Fos expression from different groups (n = 5 mice). One-way ANOVA.

Illustrations created with BioRender.com. ^*^P < 0.05, ^**^P < 0.01, ^***^P < 0.001, ^****^P < 0.0001, ns - no significant difference. Error bars indicate the SD.


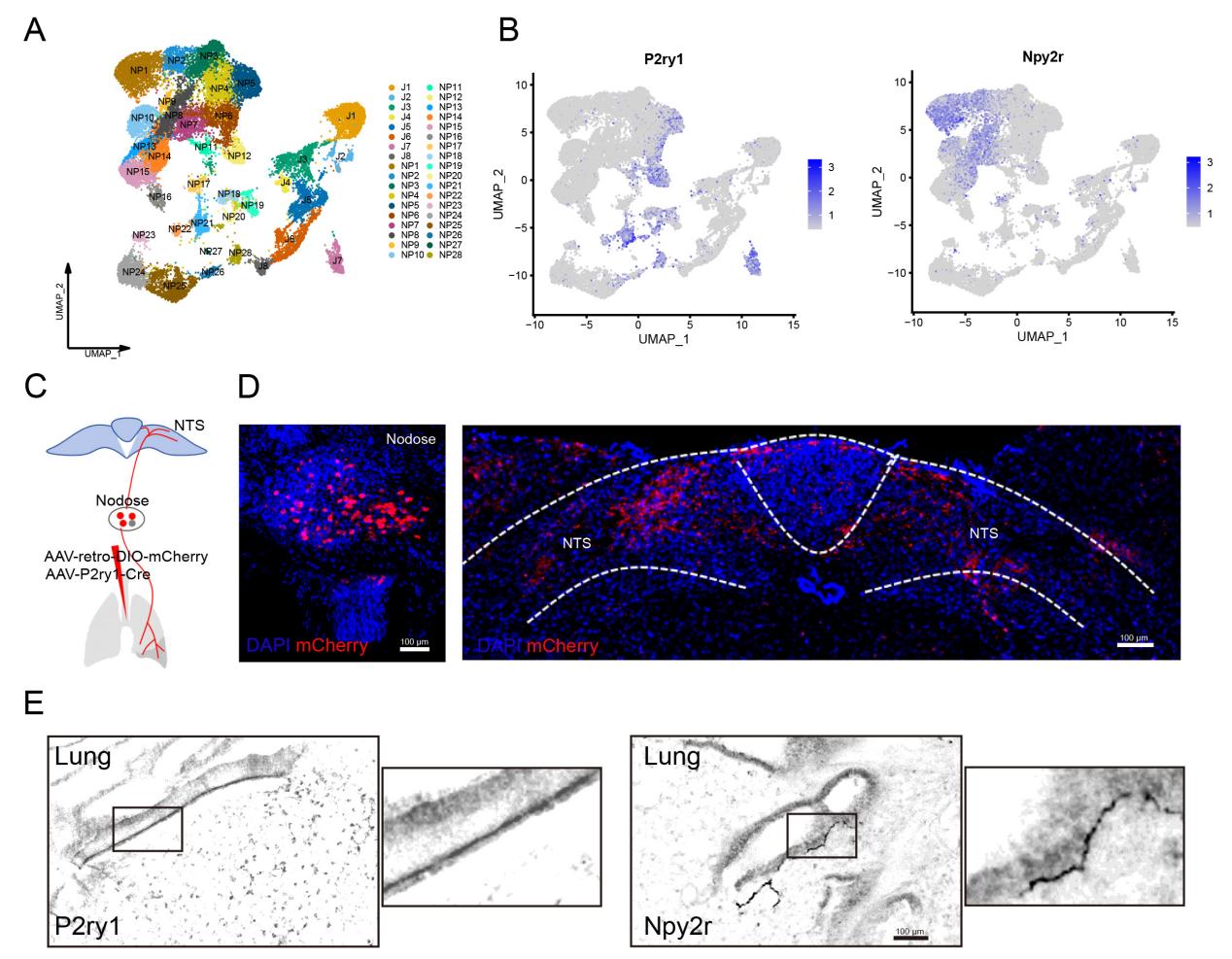


**Supplementary Figure 4. The distribution of lung-innervating *Npy2r* and *P2ry1* sensory neurons. Related to Figure 3.**

1. Uniform manifold approximation and projection (UMAP) plots indicating cell subtype diversity of vagal sensory neurons. Jugular (J1-J8) and nodose/petrosal (NP1-NP28) ganglia. Reanalysis from a previous report (GSE145216).
2. UMAP plots indicating the expression of genes (*Npy2r* and *P2ry1*) in vagal sensory neurons.
3. Schematic of neural tracing of *P2ry1* sensory neurons innervating lungs.
4. Left: mCherry-labeled *P2ry1* neurons in a nodose ganglion from three independent experiments. Right: fiber distribution of *P2ry1* sensory neurons innervating lungs in the NTS from three independent experiments. Scale bar, 100 μm.
5. Representative images showing the terminals of *P2ry1* (left) and *Npy2r* (Right) sensory neurons in lungs. Scale bar, 100 μm.

Illustrations created with BioRender.com.

**
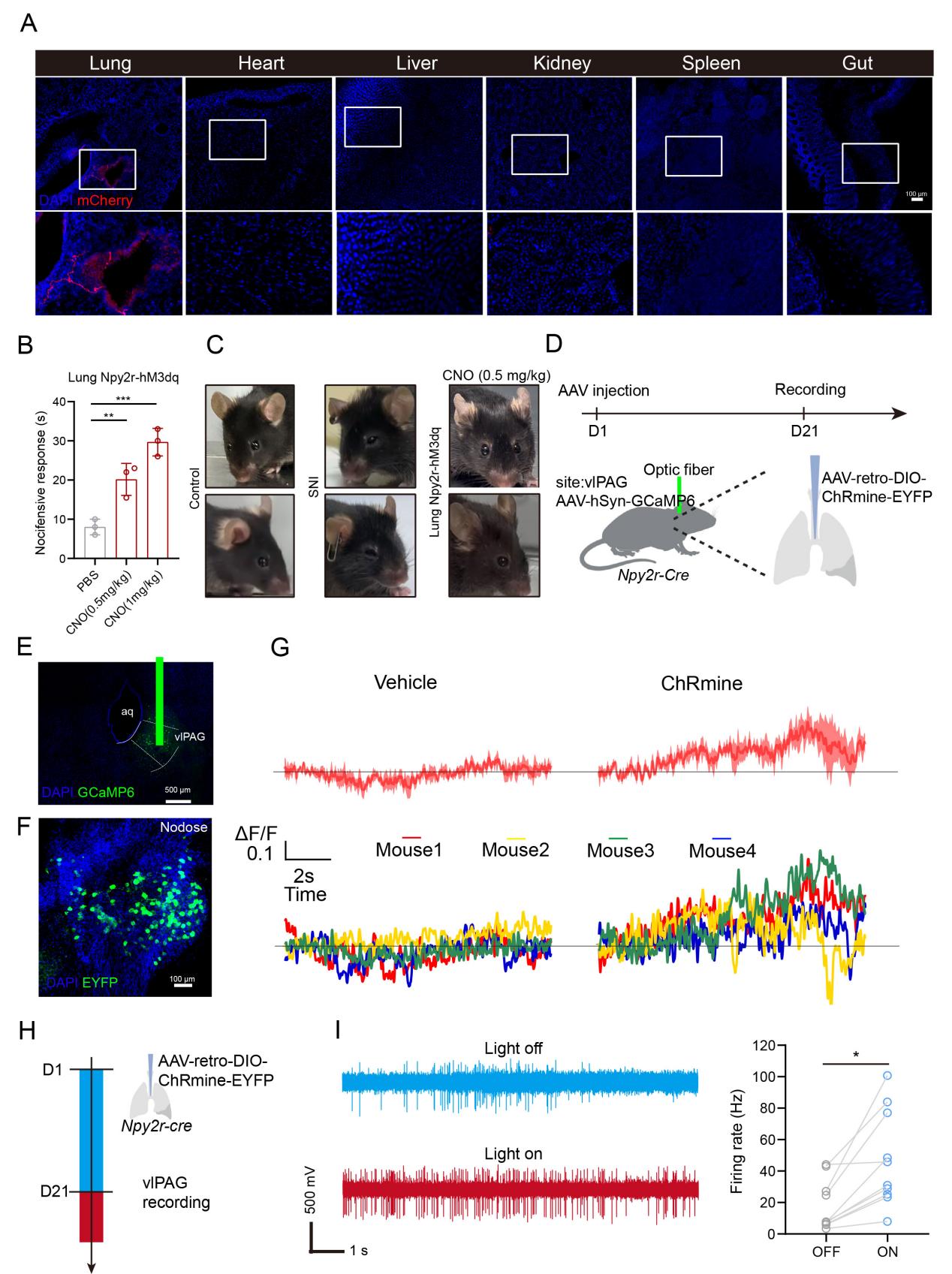
**

**Supplementary Figure 5. The activation of lung-innervating *Npy2r* sensory neurons increases vlPAG neuron activity and reduces pain. Related to Figure 3.**

1. Representative images from three independent experiments showing AAV-retro-DIO-hM3Dq-mCherry infection in different organs. Scale bar, 100 µm.
2. Paw withdrawal latency assessed by the HPT after CNO injection (n = 3 mice). One-way ANOVA.
3. Facial expressions of mice from different groups.
4. Schematic diagram of the ChRmine optogenetics stimulation.
5. Representative image showing GCaMP6 expression in vlPAG. Scale bar, 500 μm.
6. EYFP-labeled neurons in a nodose ganglion from three independent experiments (rAAV2-retro-DIO-ChRmine-EYFP was injected into the lungs of *Npy2r-Cre* mice). Scale bar, 100 μm.
7. The traces of fiber photometry of Figure 3N (n = 4 mice).
8. Schematic diagram for in vivo electrophysiological recording in vlPAG.
9. Left: representative traces of electrical activity in vlPAG after the ChRmine optogenetics-mediated lung-innervating *Npy2r* sensory neuron activation. Right: firing rates (n = 10 neurons).

Illustrations created with BioRender.com. ^*^P < 0.05, ^**^P < 0.01, ^***^P < 0.001, ^****^P < 0.0001, ns - no significant difference. Error bars indicate the SD.

**
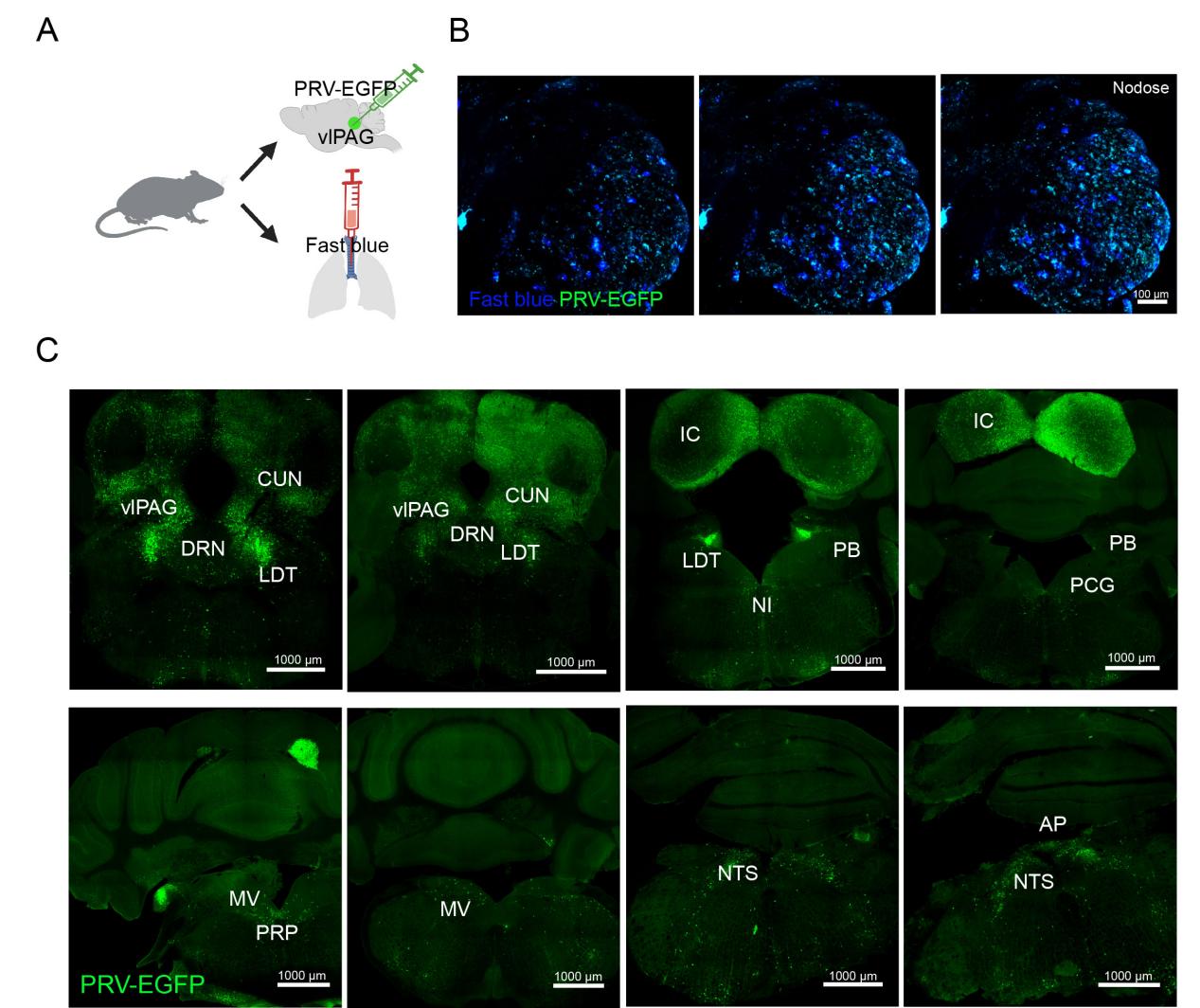
**

**Supplementary Figure 6. The neural tracing of lung-to-brain pathway. Related to Figure 3.**

1. Schematic of the neural tracing.
2. The signal of fast blue and EGFP in nodose ganglia. Scale bar, 100 μm.
3. Representative images showing retrograde labeling in brain regions between vlPAG and NTS. Scale bar, 1000 µm. vlPAG, ventral lateral parts of the periaqueductal gray area; DRN, dorsal nucleus raphe; CUN, cuneiform nucleus; LDT, laterodorsal tegmental nucleus; PB, parabrachial nucleus; NI, nucleus incertus; IC, inferior colliculus; PCG, pontine central gray; MV, medial vestibular nucleus; NTS, nucleus of the solitary tract; AP, area postrema.

Illustrations created with BioRender.com.


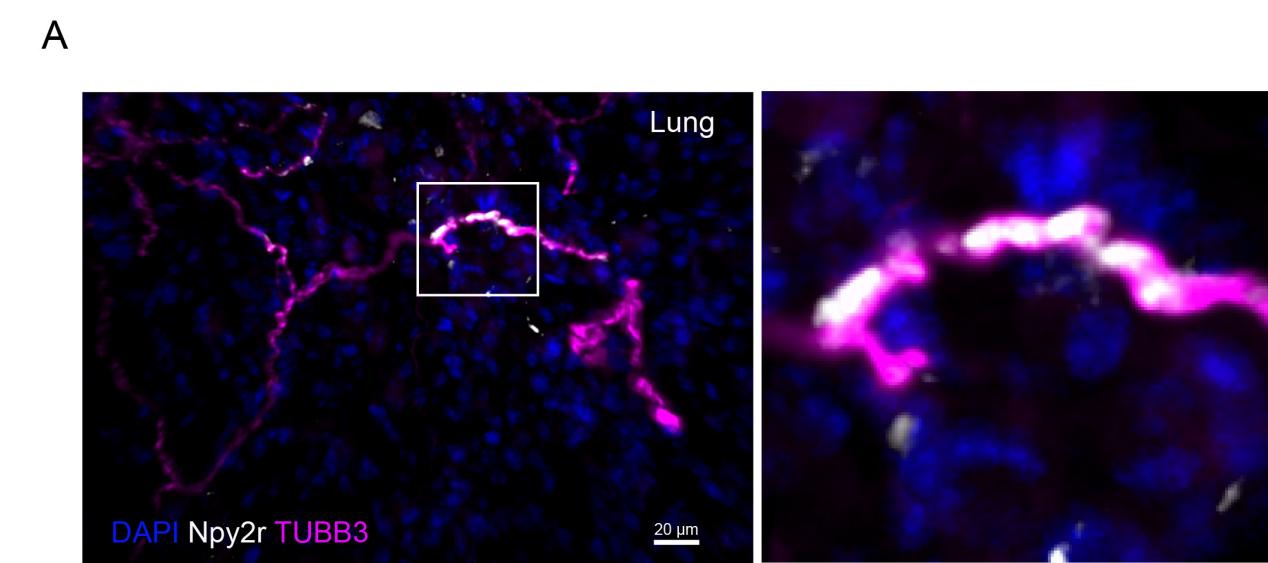


**Supplementary Figure 7. The colocalization between *Npy2r* and TUBB3 expression in lungs. Related to Figure 4.**

1. Representative images showing the colocalization between *Npy2r* and TUBB3 (neuronal marker) expression in the lungs. Scale bar, 20 μm.


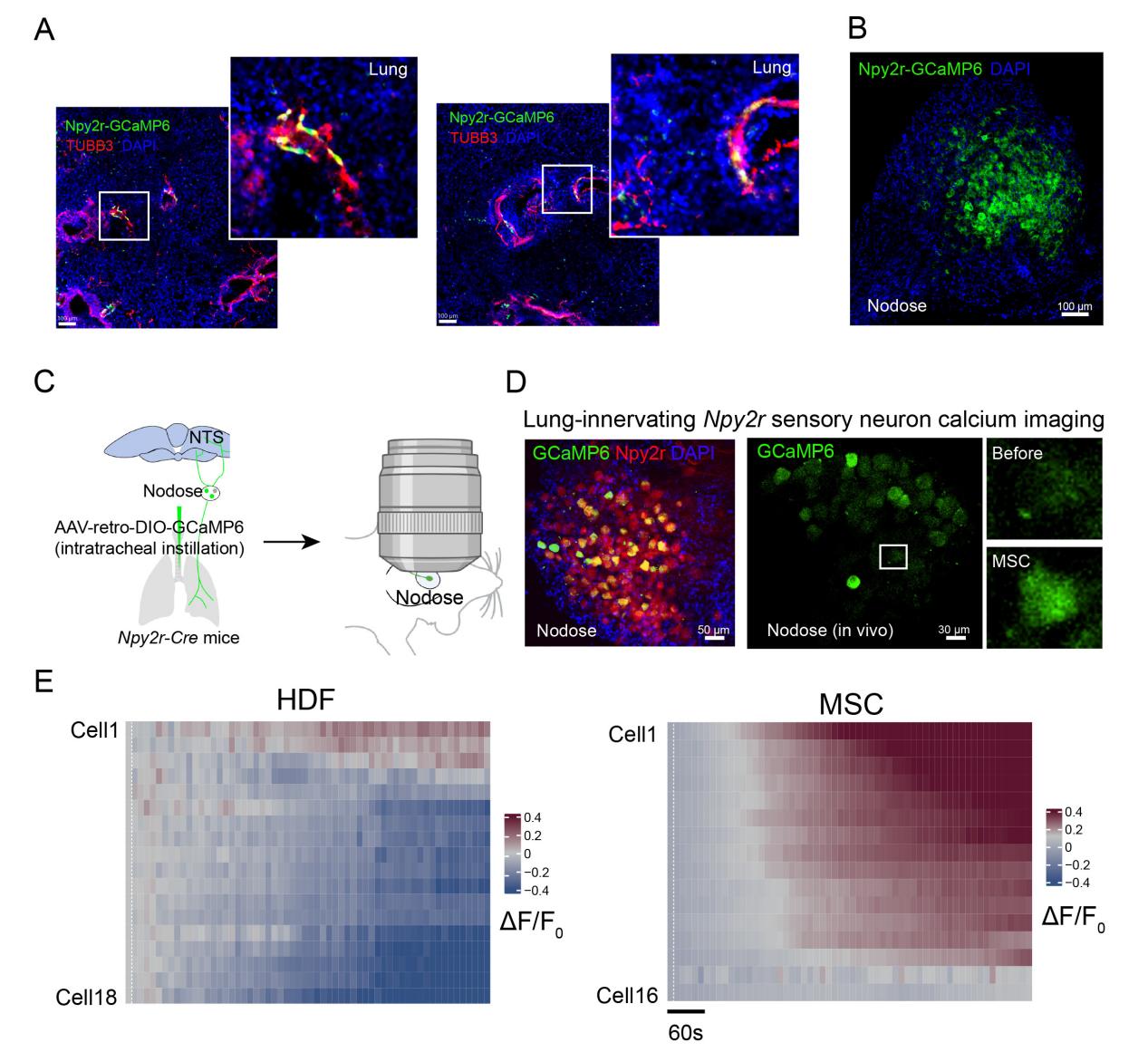


**Supplementary Figure 8. MSC activate the lung-innervating *Npy2r* sensory neurons. Related to Figure 5.**

1. Representative images showing the colocalization between Npy2r-GCaMP6 and TUBB3 in the lungs. Scale bar, 100 μm.
2. Representative images showing the Npy2r-GCaMP6 expression in the nodose ganglia. Scale bar, 100 μm.
3. Schematic diagram showing the retrograde labeling from the lungs by injecting AAV-retro-DIO-GCaMP6 into *Npy2r-Cre* mice.
4. Left: representative images showing the GCaMP6 labeling from the lungs and *Npy2r* expression in the nodose ganglia. Scale bar, 50 μm. Right: representative images showing the in vivo calcium imaging of *Npy2r* vagal sensory neurons innervating the lungs. Scale bar, 30 μm.
5. Heatmap depicting lung-innervating sensory neuron calcium responses to HDF (ΔF/F, 18 imaged neurons) or MSC injection (ΔF/F, 16 imaged neurons).

Illustrations created with BioRender.com.


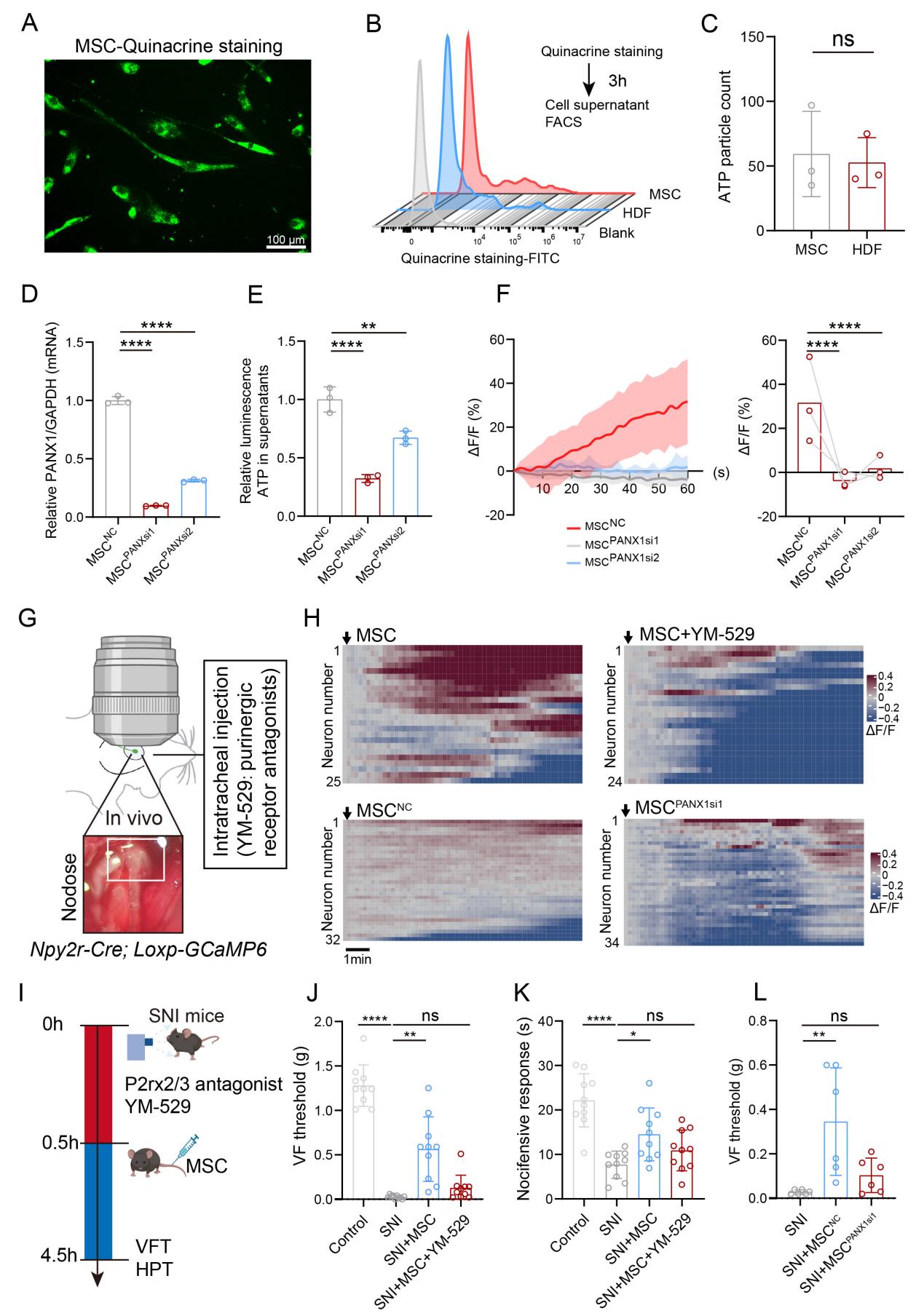


**Supplementary Figure 9. MSC exert regulatory effects on lung-to-brain axis for analgesia via PANX1-mediated ATP signaling. Related to Figure 6.**

1. Representative images showing the quinacrine staining of MSC. Scale bar, 100 μm.
2. The flow cytometric analysis of different cell supernatants after quinacrine staining.
3. Statistical analysis showing the ATP particle count in different cell supernatants after quinacrine staining. Two-tailed t-test.
4. qRT-PCR analysis confirming the siRNA-mediated downregulation of PANX1 in MSC. n = 3 biologically replicated samples. One-way ANOVA.
5. Relative ATP level in supernatants of MSC. n = 3 biologically independent samples. One-way ANOVA.
6. Left: Real-time changes in fluorescence intensity of ex-vivo lungs of *Npy2r-Cre; Loxp-GCaMP6* mice were expressed as percentage changes over baseline (ΔF/F). Right: Statistical analysis showing the peak fluorescence intensity (ΔF/F). n = 3 mice. One-way ANOVA.
7. Cartoon depicting in vivo vagal ganglion imaging after MSC or MSC^PANX1si1^ injection and YM-529 delivery.
8. Heatmap depicting *Npy2r* vagal sensory neuron calcium responses (ΔF/F, group MSC: 25 imaged neurons; group MSC+YM-529: 24 imaged neurons; group MSC^PANX1si1^: 34 imaged neurons).
9. Schematic diagram showing the procedures used for MSC treatment and YM-529 inhalation.
10. Paw withdrawal threshold assessed by the VFT. n = 10 mice.
11. Paw withdrawal latency assessed by the HPT. n = 10 mice.
12. Paw withdrawal threshold assessed by the VFT after MSC or MSC^PANX1si1^ injection. n = 6 mice.

Illustrations created with BioRender.com. ^*^P < 0.05, ^**^P < 0.01, ^***^P < 0.001, ^****^P < 0.0001, ns - no significant difference. Error bars indicate the SD.


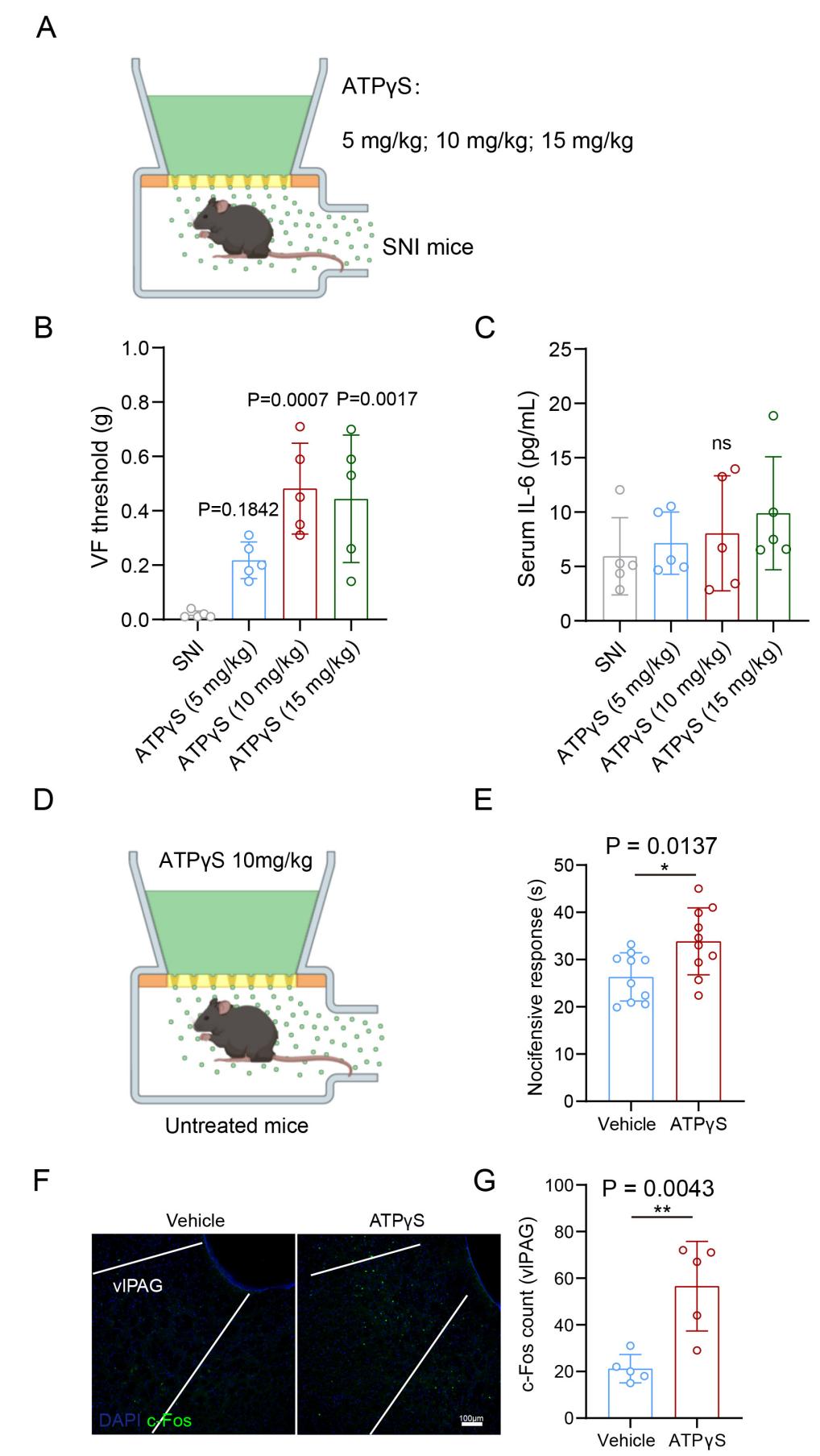


**Supplementary Figure 10. The inhalation of ATPγS improves pain threshold. Related to Figure 7.**

1. Schematic diagram showing the procedures for ATPγS inhalation in SNI mice at different dosage.
2. Paw withdrawal threshold assessed by the von Frey test (VFT) after ATPγS inhalation in SNI mice (n = 5 mice). One-way ANOVA.
3. Enzyme-linked immunosorbent assay - quantified serum concentration of interleukin (IL)-6 (n = 5 mice). One-way ANOVA.
4. Schematic diagram showing the procedures for ATPγS inhalation in non-injured mice.
5. Paw withdrawal latency assessed by the HPT after ATPγS inhalation (n = 10 mice). Two-tailed t-test.
6. Representative images showing c-Fos expression in vlPAG after ATPγS inhalation. Scale bar, 50 μm.
7. Quantification of c-Fos expression in vlPAG after ATPγS inhalation. n = 5 mice. Two-tailed t-test.

Illustrations created with BioRender.com. ^*^P < 0.05, ^**^P < 0.01, ^***^P < 0.001, ^****^P < 0.0001, ns - no significant difference. Error bars indicate the SD.

**
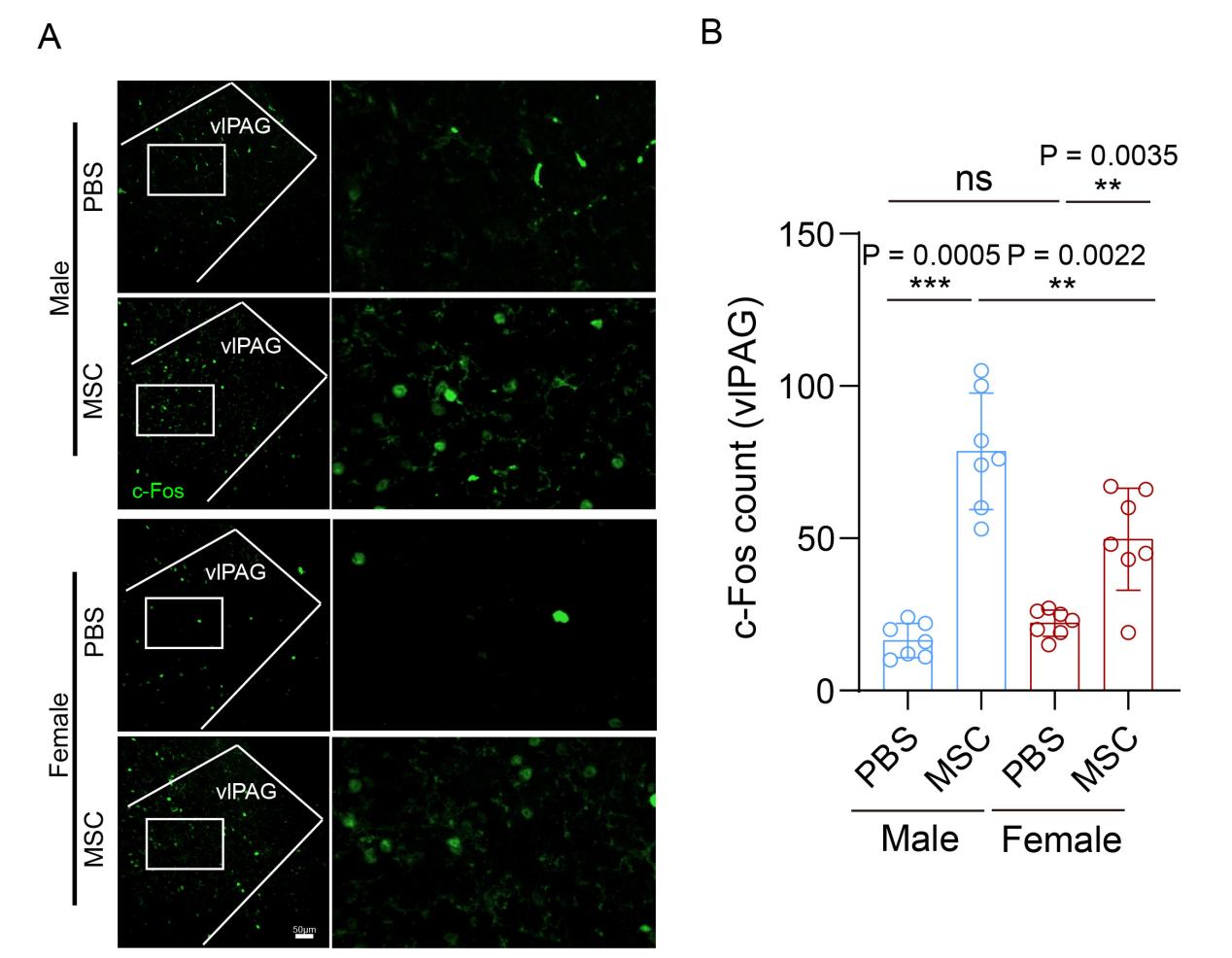
**

**Supplementary Figure 11. MSC induce the activation of vlPAG neurons in male and female mice. Related to Discussion.**

1. Representative images showing c-Fos expression in vlPAG of male and female mice after MSC injection. Scale bar, 50 μm.
2. Quantification of c-Fos expression in vlPAG of male and female mice after MSC injection. n = 7 mice. one-way ANOVA .

^*^P < 0.05, ^**^P < 0.01, ^***^P < 0.001, ^****^P < 0.0001, ns - no significant difference. Error bars indicate the SD.

**Supplementary Table 1. Antibodies**

| **Antibodies** | **Source** | **Indentifier** |
| --- | --- | --- |
| Rabbit anti-c-Fos | Abcam | Cat# ab222699; |
| Rabbit anti-Npy2r | Bioss | Cat# bs-0937R |
| Rabbit anti-TUBB3 | Abcam | Cat# ab18207 |
| PE Mouse anti-Human CD105 | BD Biosciences | Cat# 560839 |
| PE Mouse Anti-Human CD90 | BD Biosciences | Cat# 561970 |
| Alexa fluor 488 Goat anti-Rabbit secondary antibody | Invitrogen | Cat# A-21206 |
| Alexa fluor 594 Goat anti-Rabbit secondary antibody | Invitrogen | Cat# A-11012 |
| Alexa fluor 647 Goat anti-Rabbit secondary antibody | Invitrogen | Cat# A-21245 |
| Rabbit anti-PANX1 | CST | Cat# 91137S |
| Rabbit anti-GAPDH | CST | Cat# 2118S |
| Anti-rabbit IgG, HRP-linked antibody | CST | Cat# 7074S |

**Supplementary Table 2. Viruses**

| **Viruses** | **Source** | **Indentifier** |
| --- | --- | --- |
| rAAV-hSyn-DIO-taCasp3 | BrainVTA | Cat# PT-2433 |
| rAAV2-retro-DIO-EGFP | BrainCase | Cat# PT-0795 |
| HSV-EGFP | BrainVTA | Cat# H01001 |
| rAAV2-retro-DIO-mCherry | BrainCase | Cat# BC-0016 |
| rAAV2-retro-DIO-hM3D(Gq)-  mCherry | BrainVTA | Cat# PT-0037 |
| rAAV2-retro-DIO-ChRmine-EYFP | BrainVTA | Cat# PT-3669 |
| rAAV-retro-DIO-GCaMPs | BrainVTA | Cat# PT-0071 |
| rAAV-hSyn-GCaMPs | BrainVTA | Cat# PT-0145 |
| rAAV2-retro-P2ry1-Cre | BrainVTA | Cat# PT-10763 |
| rAAV2-retro-DIO-hM4D(Gi)-EGFP | BrainVTA | Cat# PT-0987 |
| PRV-EGFP | BrainVTA | Cat# P03001 |

**Supplementary Table 3. Regents and kits**

| **Regents and kits** | **Source** | **Indentifier** |
| --- | --- | --- |
| Aspirin | MedChemExpress | Cat# HY-14654 |
| Dexmedetomidine | MedChemExpress | Cat# HY-12719 |
| TUNEL kit | Servicebio | Cat# G1505 |
| Tamoxifen | Sigma-Aldrich | Cat# 10540-29-1 |
| Nuohai tissue clearing kit | Nuohai Life Science (Shanghai) | Cat# NH210701 |
| Fast Blue | Polysciences | Cat# 73819-41-7 |
| Clozapine-N-oxide (CNO) | Sigma-Aldrich | Cat# C0832 |
| DAPI | Sigma-Aldrich | Cat# D9542 |
| Luminescent ATP detection assay kit | Servicebio | Cat# G4309 |
| Minodronic acid (YM-529) | MedChemExpress | Cat# HY-16322 |
| ATPγS | Abcam | Cat# 93839-89-5 |
| LIVE/DEAD™ | Invitrogen | Cat# L34973 |

**Supplementary Table 4. Mouse strains**

| **Mouse strains** | **Source** | **Indentifier** |
| --- | --- | --- |
| Mouse: wild type C57BL/6J | Animal Center of Sun Yat-sen University (Guangzhou) |  |
| Mouse: *VGLUT2-Cre* | Jackson Laboratory | Stock No: 016963 |
| Mouse: *Npy2r-Cre* | Jackson Laboratory | Stock No: 029285 |
| Mouse: Fos^CreERT2^ | Jackson Laboratory | Stock No: 021882 |
| Mouse: Ai14 (*Loxp-tdTomato*) | Jackson Laboratory | Stock No: 007914 |
| Mouse: R26-Lck-GCaMP6f^flox^ (*Loxp-GCaMP6*) | Jackson Laboratory | Stock No: 029626 |

**Supplementary Table 5. Oligonucleotides**

| **Oligonucleotides** | **Source** | **Indentifier** |
| --- | --- | --- |
| PANX1 Primers | Sangon Biotech | 5’-CGCTGTTTGTT  CCATTCCGA-3’  5’-CCCCTGACCA  CTGCTCTTAA-3’ |
| GAPDH Primers | Sangon Biotech | 5’-GAAGGTGAAG  GTCGGAGTC-3’  5’-GAAGATGGTG  ATGGGATTTC-3’ |
| PANX1 RNAi sequences 1 | RIBOBIO | GTGAGGTCAAGTCATACAA |
| PANX1 RNAi sequences 2 | RIBOBIO | GGATCGACCCAATGCTACT |

**Supplementary sequences information**

The sequences for construction of GFP and RFP-MSC.

1. The GFP sequence 5’ - 3’

ATGGTGAGCAAGGGCGAGGAGCTGTTCACCGGGGTGGTGCCCATCCTGGTCGAGCTGGACGGCGACGTAAACGGCCACAAGTTCAGCGTGTCCGGCGAGGGCGAGGGCGATGCCACCTACGGCAAGCTGACCCTGAAGTTCATCTGCACCACCGGCAAGCTGCCCGTGCCCTGGCCCACCCTCGTGACCACCCTGACCTACGGCGTGCAGTGCTTCAGCCGCTACCCCGACCACATGAAGCAGCACGACTTCTTCAAGTCCGCCATGCCCGAAGGCTACGTCCAGGAGCGCACCATCTTCTTCAAGGACGACGGCAACTACAAGACCCGCGCCGAGGTGAAGTTCGAGGGCGACACCCTGGTGAACCGCATCGAGCTGAAGGGCATCGACTTCAAGGAGGACGGCAACATCCTGGGGCACAAGCTGGAGTACAACTACAACAGCCACAACGTCTATATCATGGCCGACAAGCAGAAGAACGGCATCAAGGTGAACTTCAAGATCCGCCACAACATCGAGGACGGCAGCGTGCAGCTCGCCGACCACTACCAGCAGAACACCCCCATCGGCGACGGCCCCGTGCTGCTGCCCGACAACCACTACCTGAGCACCCAGTCCGCCCTGAGCAAAGACCCCAACGAGAAGCGCGATCACATGGTCCTGCTGGAGTTCGTGACCGCCGCCGGGATCACTCTCGGCATGGACGAGCTGTACAAGTAA

1. The RFP sequence 5’ - 3’

ATGGATAGCACTGAGAACGTCATCAAGCCCTTCATGCGCTTCAAGGTGCACATGGAGGGCTCCGTGAACGGCCACGAGTTCGAGATCGAGGGCGAGGGCGAGGGCAAGCCCTACGAGGGCACCCAGACCGCCAAGCTGCAGGTGACCAAGGGCGGCCCCCTGCCCTTCGCCTGGGACATCCTGTCCCCCCAGTTCCAGTACGGCTCCAAGGTGTACGTGAAGCACCCCGCCGACATCCCCGACTACAAGAAGCTGTCCTTCCCCGAGGGCTTCAAGTGGGAGCGCGTGATGAACTTCGAGGACGGCGGCGTGGTGACCGTGACCCAGGACTCCTCCCTGCAGGACGGCACCTTCATCTACCACGTGAAGTTCATCGGCGTGAACTTCCCCTCCGACGGCCCCGTAATGCAGAAGAAGACTCTGGGCTGGGAGCCCTCCACCGAGCGCCTGTACCCCCGCGACGGCGTGCTGAAGGGCGAGATCCACAAGGCGCTGAAGCTGAAGGGCGGCGGCCACTACCTGGTGGAGTTCAAGTCAATCTACATGGCCAAGAAGCCCGTGAAGCTGCCCGGCTACTACTACGTGGACTCCAAGCTGGACATCACCTCCCACAACGAGGACTACACCGTGGTGGAGCAGTACGAGCGCGCCGAGGCCCGCCACCACCTGTTCCAGTAG
